# Comparison of crosslinking reagents and their impacts on bone derived ECM hydrogels

**DOI:** 10.1002/adhm.202503252

**Published:** 2026-03-19

**Authors:** Joshua N. Jones, I-Ning Lee, Andreas Rialas, Simon C. Kellaway, Rabea Loczenski, Christopher Parmenter, Lisa J. White

**Affiliations:** School of Pharmacy, University of Nottingham, Nottingham, NG7 2RD, UK; Biodiscovery Institute, University of Nottingham, Nottingham, NG7 2RD, UK; School of Pharmacy, University of Nottingham, Nottingham, NG7 2RD, UK; School of Pharmacy, University of Nottingham, Nottingham, NG7 2RD, UK; Centre for Nerve Engineering, University College London, London UK. Affiliation at time; Department of Pharmacology, UCL School of Pharmacy, University College London, London, WC1N 1AX, UK. Affiliation at time; School of Pharmacy, University of Nottingham, Nottingham, NG7 2RD, UK; School of Pharmacy, University of Nottingham, Nottingham, NG7 2RD, UK; Nanoscale and Microscale Research Centre (nmRC), University of Nottingham, Nottingham, NG7 2RD, UK; School of Pharmacy, University of Nottingham, Nottingham, NG7 2RD, UK; Biodiscovery Institute, University of Nottingham, Nottingham, NG7 2RD, UK

**Keywords:** ECM, hydrogels, crosslinking, genipin, proanthocyanidins

## Abstract

Extracellular matrix (ECM) derived biomaterials are becoming more prevalent for regenerative medicine applications as they can provide bio-instructive cues to damaged tissues, facilitating constructive remodelling of the injured site. Existing clinical products based on ECM can be found in sheet or powder formats, however recent clinical trials have utilised ECM hydrogels. Further exploration of ECM hydrogels and modulation of their mechanics, degradation and swelling behaviours could provide increased utility of these materials. Mechanical modulation through crosslinking can be achieved through application of chemical reagents such as glutaraldehyde (GA). However long-term effects of GA warrant increased research of alternatives such as the plant-based derivatives genipin (GP) and proanthocyanidins (PA). In this work, crosslinking was confirmed through alterations to the mechanical moduli of bECM hydrogels with both GP and PA conferring significantly increased moduli compared to non-crosslinked controls. *In vitro* examination determined that cytotoxic effects were observed for PA to SH-SY5Y cells at the concentration used, however no cytotoxicity was observed from PA when tested on L929 cells and primary tenocytes. There was no cytotoxicity observed from GP treatment. Therefore, use of these reagents could provide a further toolkit to manipulate physicochemical properties and increase utility of ECM hydrogels.

## Graphical abstract

**Graphical Abstract:**
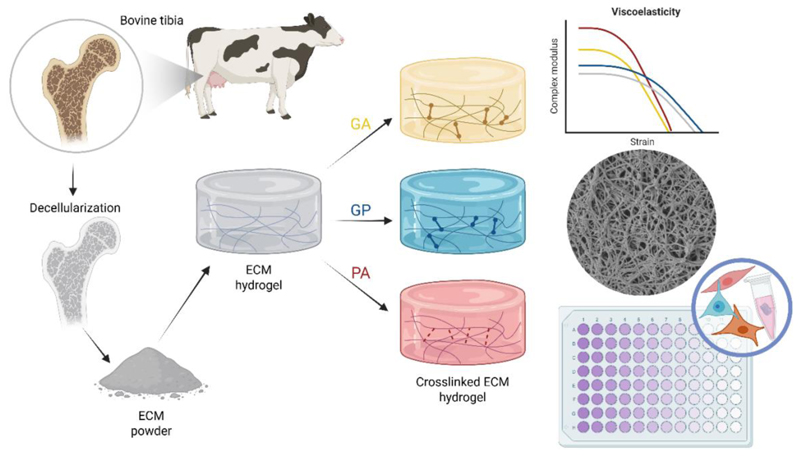

Created in BioRender. White, L. (2026) https://BioRender.com/api5efs

## 1 Introduction

Development of various decellularization techniques has facilitated generation of extracellular matrix (ECM) derived biomaterials for regenerative medicine applications.^[1–2]^ These biomaterials exploit the inherent capabilities of the ECM to exert a functional, bioinstructive effect alongside a recapitulation of the native microenvironment,^[3–4]^ thus aiding regeneration. Commercially available medical products based on ECM biomaterials have already been used clinically,^[3–5]^ often taking the format of sheets or powders, and generally used for soft-tissue repairs. However, recently a pioneering clinical trial was completed for a cardiac ECM derived hydrogel in the treatment of ventricular dysfunction.^[6]^ This foreshadows the potential inclusion of ECM derived hydrogels as clinical products. Hydrogels by nature or design are polymeric molecules incorporating large volumes of water into a three-dimensional scaffold.^[2]^ Naturally derived polymers such as collagen or gelatin can be used to generate hydrogels, with the natural interaction between fibrils producing the polymeric network. This natural gelation property was exploited by Freytes *et al*.^[7]^ to create ECM derived hydrogels, which can be obtained from numerous tissue sources.^[2]^

Levels of crosslinking within a hydrogel can be modulated at a macro or molecular scale altering the mechanical and physical properties of the hydrogel, generating stiffer, more robust materials that can also be less prone to degradation over time.^[8]^ Physically derived crosslinking mechanisms such as radiation exposure or photo-activation have demonstrated utility in hydrogel modulation, as recently reviewed by Ahmed *et al*.^[9]^ However, relatively high equipment costs and potential deleterious effects of high energy radiation exposure without sufficient optimization can limit use. Alternatively, chemical or enzymatic reagents can be employed to produce increased intermolecular linking within polymeric structures through mechanisms such as covalent or physical crosslinking, with each type of crosslinking exhibiting advantages and drawbacks.^[8, 10–11]^ Application of crosslinkers to ECM based hydrogels may further increase their clinical utility. Enhancement of ECM hydrogel strength could facilitate use in implantation sites that are exposed to increased mechanical stresses such as the compressive or shear loading within joints.^[12]^ Modulating the hydrogels degradation time could result in longer-lasting materials, to provide further time for complete integration and regeneration, or alternatively, for application in drug-delivery.^[13–14]^

Crosslinking via exogenous chemical treatment has been established as a viable methodology for ECM derived biomaterials, with clinical products such as Dura-Guard® and Peri-Guard®^[15–16]^ using glutaraldehyde (GA) for this purpose. Use of GA has been well established with ECM derived materials,^[8, 17]^ with Zhan *et al*.^[18]^ recently demonstrating GA crosslinking capabilities on decellularized placental sponges. Primarily, GA interacts with the free amino groups of lysine residues, which occurs through both monomeric and polymeric GA inclusion dependent on reaction conditions.^[19]^ This interaction results in covalent crosslinks within polymer matrices, significantly increasing strength and physical characteristics of the material.^[20–23]^

However, concerns over cytotoxicity^[21, 24]^ and calcification^[25–27]^ of GA crosslinked materials have resulted in ongoing efforts to remediate the reported toxicity of GA.^[28–29]^ Plant derived crosslinking agents such as genipin (GP) and proanthocyanidins (PA) may provide alternatives to GA. Both GP and PA have been used in traditional-medicine or nutraceutical-based therapies, with GP having anti-inflammatory, anti-bacterial and anti-neoplastic properties,^[30–31]^ and PA having cardioprotective, neuroprotective and anti-neoplastic properties.^[32–34]^

The crosslinking action of GP is similar to GA, interacting with primary amine residues, to generate covalent bonds between residues.^[35–36]^ Polyphenols, such as PA, encompass a range of polymeric flavonoids, all based on multimers of catechin, gallocatechin and afzelechin.^[37–38]^ These molecules are rich in hydroxy groups and therefore enable multiple hydrogen bonds between PA and substrate, generally involving primary amines of lysine residues and carbonyl groups of peptide bonds for collagenous materials.^[39–41]^ Both GP and PA have recently been applied to crosslink ECM biomaterials such as decellularized tissues,^[40, 42–43]^ but few studies have explored their use with ECM derived hydrogels.^[44–45]^ Bone derived ECM hydrogels exhibit increased relative strength in comparison to ECM gels derived from soft-tissue sources,^[46]^ making them ideal candidates for future clinical use.

This study for the first time establishes a standardised methodology for comparison of three different crosslinking agents, GA, GP and PA, applied to bone ECM (bECM) hydrogels. We elucidated the crosslinking mechanisms and physicochemical modulation achieved. Thorough characterisation of the physical and mechanical modifications, alongside cytocompatibility testing, demonstrates the impact of cross-linking ECM hydrogels and highlights potential applications of these bioinstructive biomaterials.

## 2 Results & Discussion

### 2.1 Decellularized bECM & hydrogel generation

Successful decellularization was evaluated through quantitative measurements of DNA and sulphated glycosaminoglycan (sGAG) quantity (Figure S1, Supporting Information). Residual double stranded DNA (dsDNA) content following decellularization was significantly decreased compared to native tissue, 21.5 ± 0.8 ng mg^-1^ (mean ± SD) of tissue and 939.3 ± 3.2 ng mg^-1^ (mean ± SD) tissue respectively (Figure S1, Supporting Information). This level of residual DNA constitutes a 98% reduction in DNA and falls below the 50 ng mg^-1^ threshold outlined by Crapo *et al*.^[47]^ Whilst DNA content was significantly reduced, sGAG content remained at 257 ± 31 ng mg^-1^ (mean ± SD) (Figure S1, Supporting Information), increasing in proportionality of ECM weight in comparison to native tissue weight. These results illustrate a successfully decellularized bECM material with retention of key functional/structural components such as sGAGs. Utilising the method developed by Freytes *et al*.,^[7]^ partial enzymatic digestion of the bECM and subsequent neutralisation yielded self-supporting bECM hydrogels ready for crosslinking (Figure S1, Supporting Information).

### 2.2 Crosslinking development

A wide variety of crosslinking agents have been explored for stabilisation of collagen hydrogels, with a recent review by She *et al*.^[48]^ highlighting studies using chemicals such as carbodiimides, alongside naturally derived reagents such as epigallocatechin gallate (EGCG) and gibberellin. Given the diversity of reagents used and subsequent differences in reaction mechanisms and conditions, this study focussed on the extensive evaluation of GP and PA in comparison to GA. Initial testing was conducted using a low concentration (0.312% w/v) of GA, GP and PA over a range of times (15 minutes, 2 hours and 20 hours) to investigate the effect of reduced crosslink time and evaluate crosslinker differences. Changes in mechanical properties were observed across all three timings, however significant differences between the three crosslinkers was observed for 2- and 20-hour crosslinking times (Figure S2, Supporting Information) with the most evident crosslinking occurring at 20 hours. Prolonged crosslinking periods are commonly used,^[49–50]^ however given the observed differences at the 2 hour crosslinking time, this was carried forward for further investigation. Using a shorter crosslinking time provides an opportunity to fine tune crosslinking effects through changes in time, concentration and reagent.

Molarity concentrations were examined as this accounts for number of molecules of crosslinking reagent per volume, reducing the effects of varied molecular weight between all three reagents (Table S1, Supporting Information). Glutaraldehyde is commonly employed in concentrations from 0.5-0.625 %w/v^[17, 20–22]^ equating to approximately 50 and 62 mM. This far exceeds the concentrations generally explored for GP and PA investigations, impeding direct comparisons. Therefore, concentrations of 10 and 50 mM were initially evaluated for concentration dependent effects.

**Figure 1:**
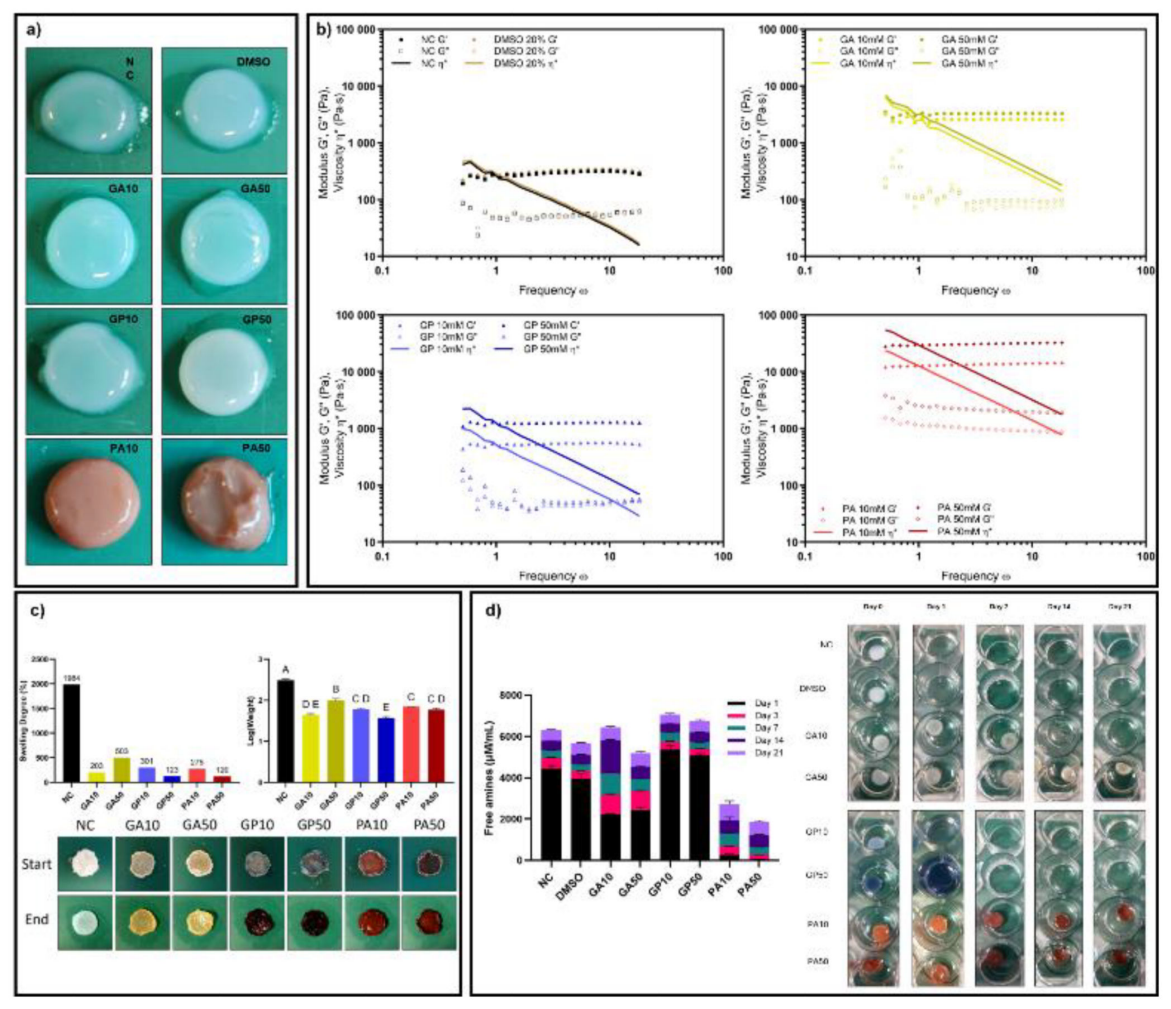
(a) Representative imaging of 10 and 50 mM crosslinked bECM hydrogels, images not to scale; (b) Rheological frequency sweeps of crosslinked gels. N=3, n=3; (c) Assessment of gel swelling following crosslinking with representative images [not to scale]. Data presented as mean ± SEM, N=3, n=2. Significance determined through one-way ANOVA with shared lettering dictating non-significant (p>0.5) groups; (d) Enzymatic degradation of crosslinked gels quantified through ninhydrin reaction of digest eluant, with representative images [not to scale]. Data presented as mean ± SEM, N=3, n=2.

Addition of crosslinking reagents altered the colour of the hydrogels, with GA yielding a slight yellow colouration, GP producing slight blue pigmentation, and PA making the hydrogels red and opaque, with no visible changes resulting from DMSO exposure (Figure 1a).

The observed red colouring of the PA crosslinked gel was consistent, with intensity increasing in line with PA concentration, with that observed by Zhai *et al*.^[42]^ in crosslinked decellularized veins. Opacity caused by PA crosslinking is potentially due to the proposed hydrophobic binding interactions between PA and proline residues ^[41, 51–52]^ within the collagen component of the bECM. These hydrophobic interactions may lead to a level of colloidal turbidity due to water leaving the system, with increases in hydrophobicity of PA treated collagen films being demonstrated by He *et al*.^[49]^

Interactions between GP and primary amines resulted in a blue colouring,^[36]^ which is often used as a metric for crosslinking success.^[50, 53]^ Extended reactions times are commonly reported, which may account for blue colouring at lower concentrations,^[50, 53]^ however this was not observed in this study. Throughout this study, blue colouring of GP crosslinked hydrogels was erratic and could be attributed to the multi-step formation of GP pigments.^[54–55]^

Increases in rheological properties of the crosslinked hydrogels were observed (Figure 1b). Compared to non-crosslinked (NC) controls there were significant increases in storage moduli for all crosslinked gels corresponding to 8- and 10-fold increases in storage modulus for GA concentrations, 1.8- and 4.2-fold increases for GP, and 39- and 89-fold changes for PA (Figure 1b).

Crosslinked hydrogels exhibited a significant increase in physical stability upon examination of swelling degree (Figure 1c). Non-crosslinked gels demonstrated a marked increase in weight following drying and subsequent rehydration, with a swelling degree of 1984%. All crosslinked gels ended with swelling degrees of 500% or less, significantly lower than NC gels (Figure 1c). The DMSO treated gels disintegrated during testing (data not shown). This is potentially due to the lycoprotective effects of DMSO altering ice crystal formation and inducing structural differences following lyophilisation,^[56]^ leaving the gel unable to withstand the deformational stresses of swelling.

Relative increases in gel stability also resulted in pronounced degradation resistance, particularly for GA and PA treated gels (Figure 1d), as shown previously.^[22, 43]^ There was an observed concentration dependency within the GA treated gels, with GA10 samples degrading within 2 weeks whilst GA50 samples persisted for the full 3-week timepoint (Figure 1d). However, no changes were observed in PA treated samples for either concentration over the complete time course. In conjunction with the aforementioned hydrophobic interactions of PA and proline in collagen,^[41, 51–52]^ Petelski *et al*. postulated the ability for PA oligomers to interact with proline residues by wrapping around them,^[41]^ and therefore we hypothesise that this potential interaction could shelter the collagenase target sequence (proline-X-glycine-proline motif) from the enzyme, thus inhibiting its action and sample degradation.^[57–58]^ Of note was the complete dissolution of both GP10 and GP50 samples within 24 hours.

Whilst increased mechanical moduli and hydrogel stability could provide increased utility, the observed lack of degradation in the 50 mM crosslinked samples could lead to undesirable effects such as a foreign body response, resulting in fibrosis and material encapsulation.^[59–60]^ Further to this, qualitative examination of the crosslinking solutions and the PBS washes revealed an identifiable presence of residual crosslinker following washing (Figure S3, Supporting Information), with a concentration dependence. It was reasoned that reductions in crosslinker concentration would be preferable, with decreased concentrations demonstrating less residual crosslinker after the wash protocol (Figure S3, Supporting Information). Thus, only 1- and 10-mM concentrations were carried through for further study.

### 2.3 Crosslinked bECM hydrogel characterisation

#### 2.3.1 Inclusion of crosslinker

**Figure 2:**
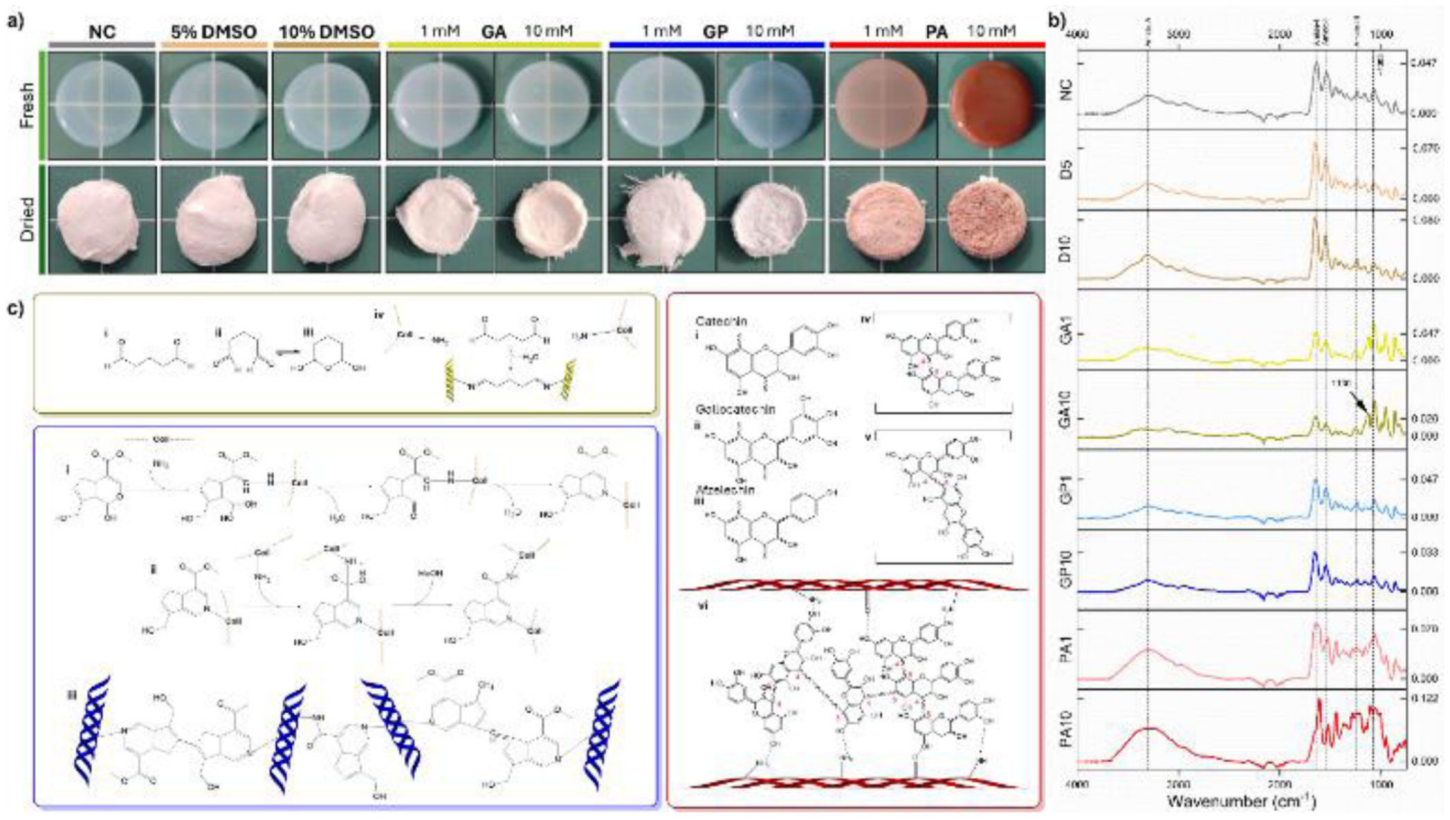
(a) Representative images of crosslinked bECM hydrogels in fresh and freeze-dried formats; images not to scale; (b) FTIR spectra of crosslinked bECM hydrogels, y-axis scale values represent baseline and absorbance value for peak 1650 cm^-1^; (c) proposed crosslinking mechanisms for GA [yellow box], GP [blue box], and PA [red box].

Colour changes to crosslinked gels at reduced concentrations mirrored those observed prior, with noticeable concentration dependent effects (Figure 2a). Confirmation of crosslinker integration into the bECM gel structure was investigated through FTIR spectroscopy and ninhydrin quantification.

Infrared spectroscopic evaluation demonstrated the characteristic peaks of collagen, including amide I (~1650 cm^-1^, C=O stretching), amide II (~1550 cm^-1^ N-H bending and C-N stretching), amide III (~1240 cm^-1^, C-N stretching, N-H plane bending, CH_2_ wagging), and amide A (~3350 cm^-1^, N-H stretching, influenced by hydrogen bonding), with a peak at ~1083 cm^-1^ indicating a fibrillar structure.^[61]^ No discernible changes in spectra between NC samples and DMSO controls was observed (Figure 2b), alongside no large-scale changes to bandings from addition of GA and GP into the bECM hydrogel. This contrasts with the findings of Sundararaghavan *et al*.,^[62]^ however this is likely due to a reduced crosslinking time (2 hours) in this study. Of note, GA samples had a new peak present at ~1130 cm^-1^ attributed to the C-O stretching of ether bonds, indicating the potential presence of hemiacetal GA polymers which can be present in aqueous environments.^[19, 63]^ Addition of PA into the hydrogel resulted in the distinct broadening and slight shift of the amide A peak indicating increased levels of hydroxyl groups such as those found in PA, as well as increased levels of hydrogen bonding, consistent with a recent study by Hidalgo-Vicelis *et al*.^[44]^ Alongside this were new peaks at 769 and 824 cm^-1^ (C-H bending), 1004 cm^-1^ (C-O stretching) and 1107 cm^-1^ (aromatic C-C stretching), 1610 cm^-1^ (aromatic C=C bending), 3214 cm^-1^ (aromatic CH stretching), further indicating increased presence of PA molecules.

Inconclusive evidence of amine group consumption was observed through FTIR investigation for GA and GP, therefore, quantification of amine content through a ninhydrin-based assay was undertaken. Incubating crosslinked bECM hydrogels in ninhydrin solution resulted in development of Ruhemann’s purple colouring, with colouring reduced in GA and GP crosslinked samples (Figure S4, Supporting Information), indicating consumption of primary amine residues. Quantification of ninhydrin reaction was attempted, however entrapment of Ruhemann’s purple within hydrogels resulted in unreliable measurement.

These results confirm the proposed crosslinking mechanisms outlined in Figure 2c. Treatment of GA and GP resulted in visually reduced levels of amine content, whilst presence of the ~1130 cm^-1^ peak in GA treatment indicated presence of polymeric forms of GA inclusion. Use of PA resulted in increased hydrogen bonding within the sample; however further assessment could provide more information on oligomeric or polymeric inclusions.

#### 2.3.2 SEM Imaging

**Figure 3:**
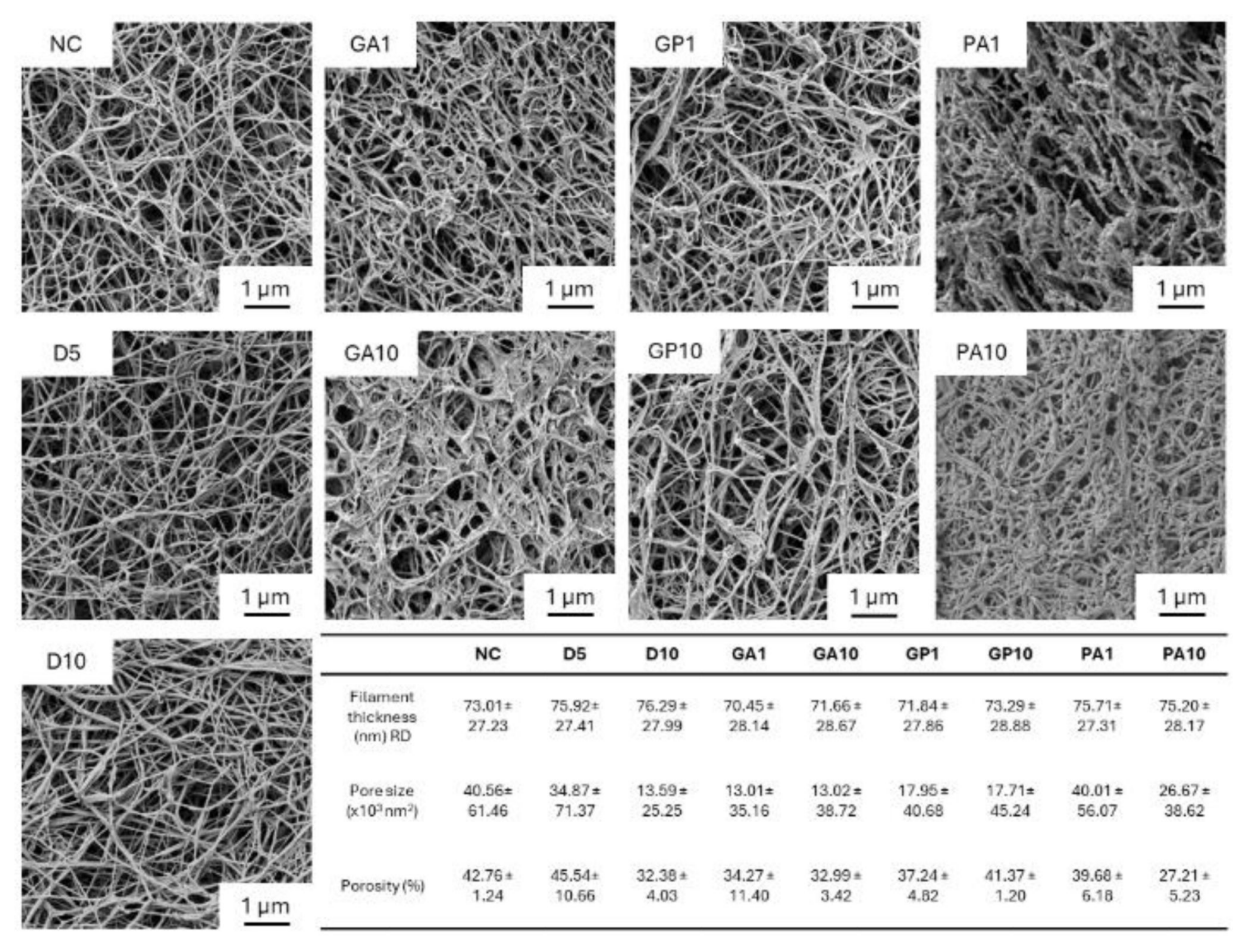
Representative SEM images of crosslinked bECM hydrogels (magnification, x10k) and physical measurements.

Changes to gel morphology following crosslinker treatment can be seen in Figure 3. All samples demonstrated a typical fibrous matrix ultrastructure, with random orientation of collagen fibres, and no apparent large-scale changes to fibre thicknesses following crosslinking. However, visual changes were apparent in crosslinked gels, with pronounced webbing effects observed from GA and GP treatments increasing in line with concentration, potentially indicating presence of inter-molecular crosslinks between collagen fibres, promoting cross strand interactions. This was corroborated by decreases in surface porosity and pore area of the GA10 and GP10 samples. Reductions in porosity was also observed in PA10 samples; however, no webbing effects were present, with crosslinking promoting a more comprehensive, matted effect. Notable changes following PA exposure was the presence of rounded particulate matter across collagen fibres, potentially indicating the presence of excess, insoluble PA deposited on the hydrogel. Observations across all samples, with exception of GA10 and PA10, demonstrated pronounced fibre breaking, likely due to sample freezing and preparation. The expected increases in gel strength following GA and PA treatments would likely have contributed to their resilience and resistance to fibre breakage during processing. Preliminary assessment of network density demonstrated increases in network junctions with increases in crosslinker concentration, however further evaluation is necessary (Table S2, Supporting Information).

#### 2.3.3 Mechanical modulation

**Figure 4:**
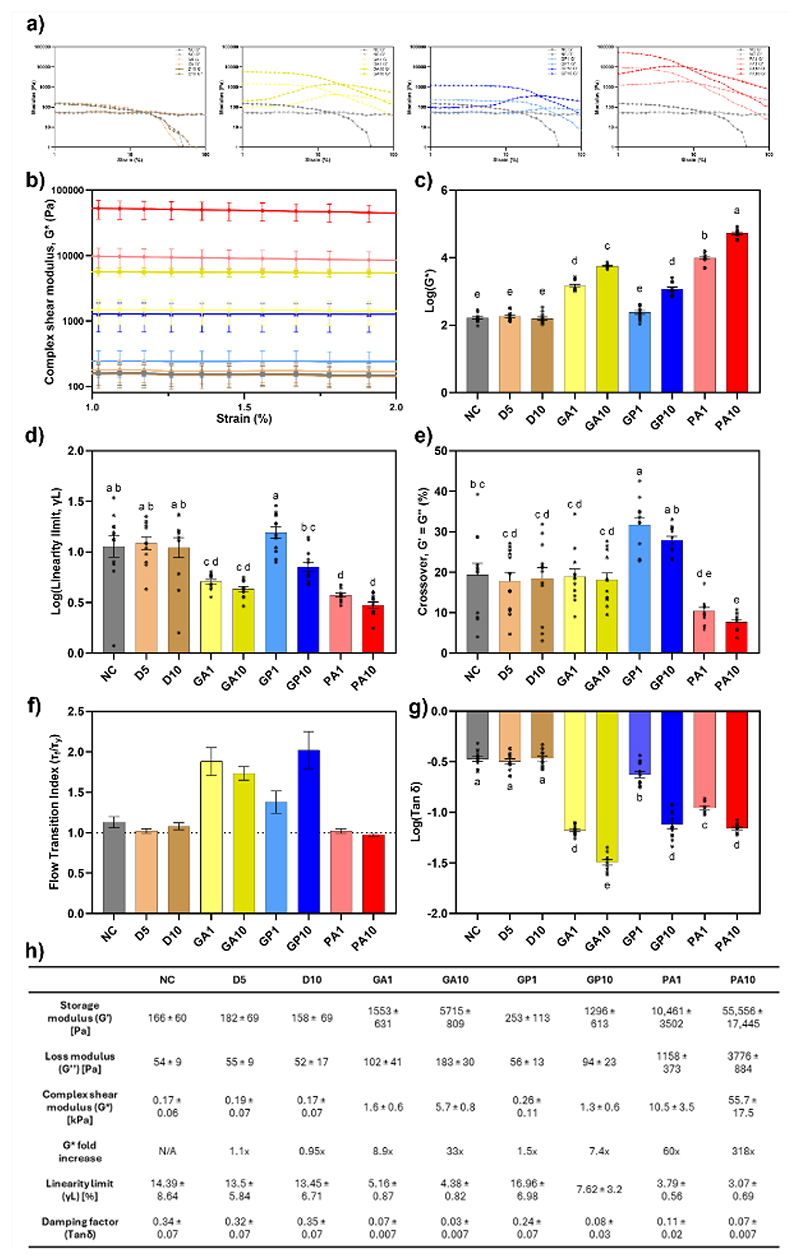
(a) Amplitude sweep profiles of control and crosslinked hydrogels; (b) Complex shear modulus profile within linear strain range; (c) Log transformed complex modulus values at 0.5% strain rate; (d) Log transformed values of maximum strain % for linear viscoelastic region; (e) Strain rate % at G′ and G″ crossover point; (f) Flow transition indices of crosslinked gels; (g) Log transformed values of damping factor tan δ; (h) Table of absolute data values for rheological properties. All data presented as mean ± SEM of N=3, n=4. Significance determined through one-way ANOVA with threshold of p<0.5, with shared lettering denoting statistically homogenous, non-significant groups.

An overview of rheological parameters evaluated can be found in Section 4.5.3. Amplitude sweeps illustrated marked upward shifts in storage (G′) and loss (G″) moduli for all crosslinked samples except GP1 samples, indicating increased ability for the crosslinked hydrogels to store elastic energy (Figure 4a, h). Storage modulus values achieved for GP crosslinking were consistent with those reported for collagen gels by Robinson *et al*. ^[64]^, whilst exceeding those achieved by Výborný *et al*.^[65]^ for their GP crosslinked ECM gels. The G′ values achieved in this work for PA crosslinking far exceeded those reported elsewhere, with Hasanzadeh et al ^[43]^ reporting a highest value of 100 Pa for composite collagen, fibrin and decellularized Wharton’s jelly hydrogels, although lower concentrations of hydrogel were used in their study. An upward trend in G* was clearly visible within the non-deformational strain range (Figure 4b). Using the G* values at a strain value of 0.5%, significant increases were observed in the order of PA10>PA1>GA10>GA1>GP10 (55.7 ± 17.5, 10.5 ± 3.5, 5.7 ± 0.8, 1.6 ± 0.6 and 1.3 ± 0.6 kPa, respectively, mean ± SEM) in comparison to NC gels (0.17 ± 0.06 kPa) (Figure 4c,h). Samples treated with GP1 (0.26 ± 0.11 kPa), along with the 5% and 10% DMSO controls (0.19 ± 0.07 and 0.17 ± 0.07 kPa, respectively) exhibited no significant changes in G* compared to NC samples (Figure 4c,h). Concentration dependent effects were observed for all three crosslinkers, with the effects of 10 mM concentrations significantly higher than the 1 mM within reagent groups.

During test periods of low strain, samples exhibited typical behaviours where the stress response is proportional to the increased strain, thus forming the linear viscoelastic region (LVR). However, the significant increases in shear moduli of crosslinked samples correlated with reductions in maximum strain endured by samples, termed the linearity limit (γL) or yield point (τ_y_) (Figure 4d). Significant reductions were observed in both GA and PA crosslinked samples for both concentrations. Whilst GP10 demonstrated lower γL values than NC, there was no significant reduction, however it was significantly reduced compared to GP1, which itself demonstrated no difference to NC gels.

Alongside changes to the limit of LVR, the crossover point was also affected through crosslinking, occurring at significantly lower strain values than all other samples for PA10, with PA1 lower than NC, but not DMSO or GA (Figure 4e). Interestingly, the GP treated samples exhibited increased values for crossover point, indicating a slower breakdown behaviour than other gels (Figure 4e).

When evaluating the flow transition index of samples, values approaching 1 denote a brittle fracturing behaviour during breakdown. Transition indices for non-crosslinked gels, as well as PA treated samples, approximated 1 (Figure 4f) indicating quick sample deformation and destruction. However, the hydrogels crosslinked with GA and GP demonstrated increased transition indices, indicating the need for much higher stresses to facilitate gel flow (Figure 4f). This behaviour may be attributed to the lengthening of polymer chains within the structure, whilst the GA and GP intermolecular crosslinks hold the chains together and impede their propensity to flow, until sufficient stress is experienced to fracture these links.

Crosslinking with all reagents and concentrations resulted in significantly reduced tanδ values (Figure 4g,h), including for GP1, despite its non-significant changes in shear moduli. This describes an increased solid behaviour within all gels following crosslinking, which was further compounded by concentration, with all reagents demonstrating significant differences between 1 and 10 mM (Figure 4g,h).

Crosslinking provided significant increases in mechanics of the bECM hydrogels, with much higher stresses being endured following crosslinker treatment. However, stiffening of the gels to behave in a more solid fashion comes at a detriment to their ability to cope with shear deformational forces. Despite this, the G* values for GA and GP crosslinked hydrogels align within the shear moduli ranges of a variety of tissues, including skeletal muscle (5-170 kPa), kidney (4-8 kPa), cardiac (5-50 kPa), thyroid (1.3-1.9 kPa), and spleen (15-20 kPa).^[66]^ The drastic increase following PA10 treatment also aligns to mechanically resilient tissues such as bladder at 50-100 kPa^[66]^; this indicates a potential area of application for cross-linked ECM hydrogels where ECM sheets are currently used.^[40]^

#### 2.3.4 Swelling Test

**Figure 5:**
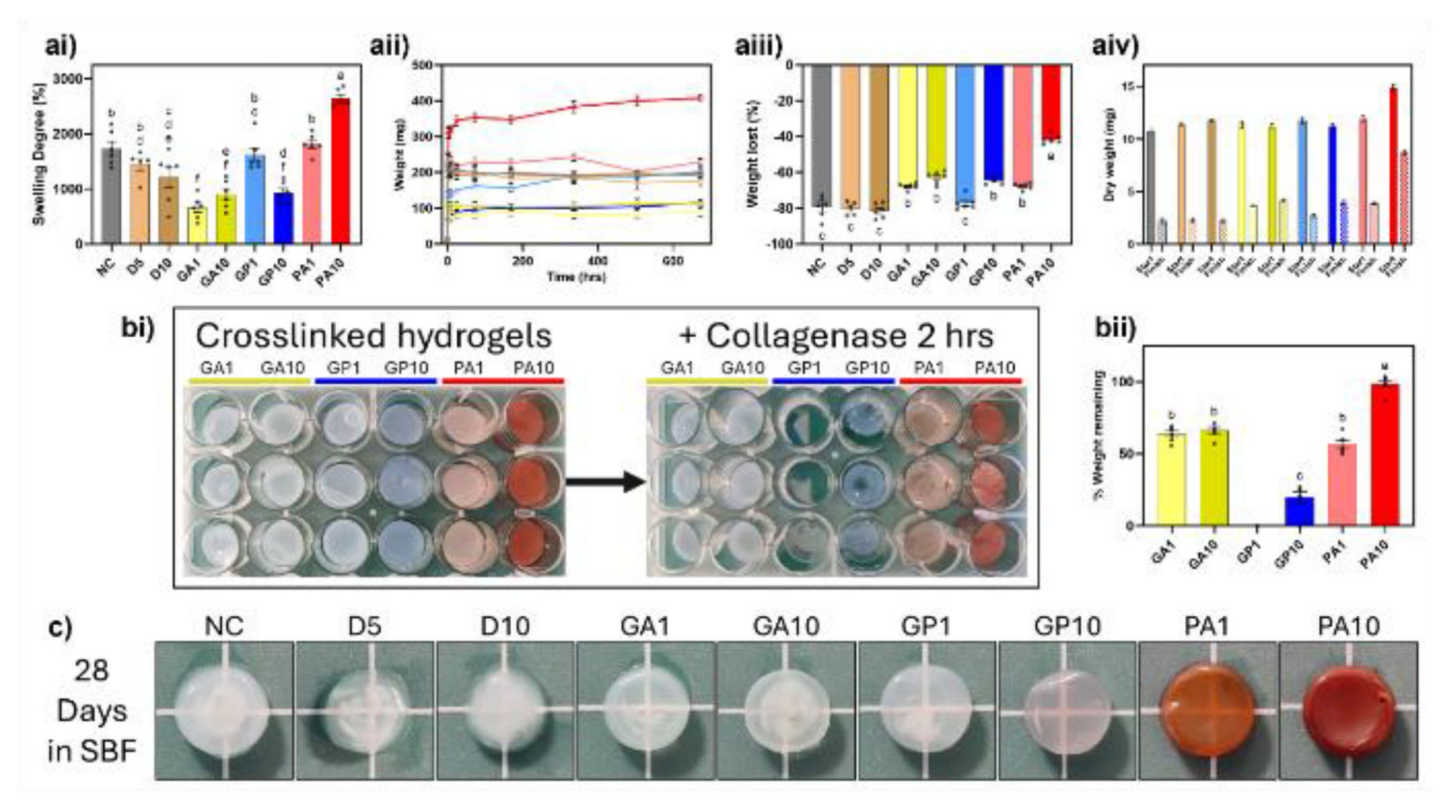
(a) Assessment of swelling behaviour of crosslinked gels including (i) swelling degree, (ii) absolute weight values over time, (iii) overall weight lost, and (iv) start and finish weight comparisons. N=2, n=3, significance determined through one-way ANOVA with shared letters denoting statistically homogenous groups; (b) Enzyme degradation resistance observed through representative images (i) and remaining gel weight after 2-hour enzyme exposure (ii). N=2, n=3; (c) representative images [not to scale] of crosslinked gels following incubation in simulated body fluid (SBF). All data presented as mean ± SEM. Significance for all assessments determined through one-way ANOVA with a threshold of p<0.5, with shared letters denoting statistically homogenous groups.

Hydrogel swelling behaviour can infer structural stability, influence mechanical profiles and dictate factors such drug release kinetics. The hydrophilic and amorphous nature of hydrogels facilitates continual uptake of water whilst crosslinking will stiffen the structure and inhibit this.^[67]^ There was no observed difference in swelling ratio or gel weights following treatment with 1 mM GP (Figure 5 ai). Crosslinking with GA (1 and 10 mM) and GP10 resulted in a significantly reduced swelling ratio in comparison to NC controls. This could be attributed to factors such as the reduced pore size inhibiting uptake or decrease in hydrophilicity due to consumption of NH_2_ groups through crosslinking mechanisms.^[67]^ Whilst pore sizes were also reduced for GP1 samples, continued uptake of solution over the time course of the experiment resulted in a non-significant swelling ratio compared to NC. All gels exhibited drastic increases in weight within hours of rehydration, with weight gain in non-crosslinked controls (NC, D5, D10), GA gels (1 and 10 mM) and PA1 gels effectively plateauing following this initial increase (Figure 5 aii; Figure S5, Supporting Information). Despite this plateau, both GA samples demonstrated a lower overall weight increase throughout the experiment compared to control gels. This indicated increased gel stability with strengthened architecture resisting deformational stresses of swelling and inhibiting further uptake. The GP and PA10 crosslinked samples demonstrated consistent increases in weight throughout the time course of the experiment (Figure 5 aii; Figure S5, Supporting Information), potentially demonstrating an increase in pliability. The PA10 samples demonstrated the highest weight gain and subsequent swelling ratio of all samples, with seemingly little effect on physical gel appearance (Figure S5, Supporting Information).

Evaluation of the final dried weight of the samples allowed for measurement of loss through hydrolysis (Figure 5 aiii,iv), with all samples losing weight through the course of the experiment. The PA10 samples demonstrated the least amount of lost weight; the overall trend corresponded to the inverse of the swelling degree. This provides further confidence in the strengthening of the gel structure following crosslinking treatments, resisting hydrolytic breakdown and dissolution over time.

Of note was the swelling behaviour exhibited by the PA1 samples. Throughout the experiment, PA1 samples mimicked the trends of non-crosslinked control samples, demonstrating vast uptake of PBS within hours or addition, followed by a plateau, with weight values similar to controls. These behaviours resulted in non-significant difference in swelling ratio, as well as marked increase in weight lost compared to PA10 samples. Upon physical handling, PA1 gels were soft with a liquid-like internal structure (Figure S5, Supporting Information), which was not seen in any other sample. Through these observed changes, it was hypothesised that there was a non-crosslinked core present within the PA1 treated samples. The potential problematic penetration of PA into hydrogels has been well documented,^[39, 43]^ and may be due to the large, polymeric molecules of PA and interaction with collagen hindering diffusion into the core of the sample.

#### 2.3.5 Degradation Resistance

Degradation of implanted ECM constructs is crucial in facilitating the beneficial constructive remodelling response and enhancing material integration.^[3]^ Given the extended resistance to enzymatic degradation observed from 10 and 50 mM crosslinked gels, collagenase concentration was increased to provide an accelerated degradation timeframe as per the protocol outlined by Výborný *et al*.^[65]^ Overall degradation trends in 1 and 10 mM crosslinked gels demonstrated concentration dependent effects, with overall resistance in order of PA>GA>GP. Following 2 hours of incubation in collagenase enzyme, control samples were completely solubilised. The GP1 samples also degraded within this timeframe (Figure 5 bi), differing to reported results despite similar gel maturation times and collagenase exposure.^[65]^ This could illustrate potential differences in GP effect depending on use as an external solution as in this work, or direct inclusion into the gel.^[65]^ Assessment of crosslinking degrees via ninhydrin assay between GP gels crosslinked through inclusion or external exposure could verify if crosslinking variances exist, however reliability concerns over ninhydrin quantification within this study (Figure S4, Supporting Information) hindered assessment. There were also visible degradation effects on GP10 and PA1 samples, with apparent defects in gel structure, with the gels losing an average of 80% and 43% of their weights respectively (Figure 5 bii). The GA treated samples also lost large volumes in weight, with average losses of 36% and 34% for 1 and 10 mM respectively, a non-significant difference. The PA10 treated samples proved resilient against degradation, retaining 99% of their initial mass, significantly higher than all other samples.

The PA1 samples had pronounced degradation within a short timeframe. Visible changes in the gels illustrated reduced colouring and increased transparency within the middle of the treated gels (Figure 5 bi), indicating digestion of the internal structure and possible presence of a non-crosslinked core. Diffusion of PA into the hydrogel was observed to be concentration dependent, with increased concentration displaying the characteristic bulking of collagen fibres internally (Figure S5, Supporting Information). It is possible that lower concentration (1 mM), reduced reaction time (2 hours), and poor aqueous solubility of PA all contributed to insufficient diffusion for thorough crosslinking, resulting in a non-crosslinked core. Cross-sectional assessment of GA and GP gels illustrated webbing effects of crosslinker internally, likely due to smaller molecular size and increased solubility facilitating diffusion and crosslinking (Figure S6, Supporting Information).

#### 2.3.6 Anti-calcification effects

Reported calcification of GA crosslinked implants^[25–27]^ facilitates investigation of other reactive compounds such as GP and PA for modulation of materials. In conjunction with this, ECM biomaterial use is driven by the bioactive capabilities of the native tissue remaining within the ECM, exerting their bio-instructive cues and facilitating regeneration.^[3]^ Given the derivation of our ECM hydrogels from mineralised bone tissue alongside crosslinking effects, it is imperative to estimate levels of calcification within our samples.

After 1 month in simulated body fluid (SBF), control gels (NC, D5, D10) demonstrated presence of a white precipitate within the gel (Figure 5c). Staining by alizarin red confirmed this precipitate to be calcium based, indicating mineralisation of the gel core (Figure S7, Supporting Information). Minor mineralisation was observed in GA1 gels, but there was a marked visual increase of precipitate in GA10 gels (Figure 5c), aligning with reports of increased calcification of GA samples.^[25, 68]^ Mineralisation was much decreased in GP samples compared to controls, with a further reduction in conjunction with increased GP concentration (Figure 5c). Further still, there was no observed precipitate within any PA crosslinked sample, however the increased opacity of PA10 gels prevented inspection of gel internal structure. These results align with published reports assessing anti-mineralisation effects of GP and PA on decellularized or collagenous constructs, for both *in vitro* and *in vivo* experimentation.^[39, 69]^

### 2.4 Cytocompatibility testing

Current progress of ECM derived hydrogels and the potential advent of their clinical use may open avenues for a new generation of ECM gel-based biomaterials. Whilst using ECM materials in a site homologous to tissue source can be seen as optimal, it is not necessary to restrict use and shy away from heterologous use, with numerous ECM products already used clinically in heterologous sites, as discussed by Cramer *et al*.^[3]^ Recent work by Kellaway *et al*.^[46]^ demonstrated the feasibility of bECM hydrogels for use in nerve conduit repair. Further modulation of bECM gels by GP and PA may provide stronger, degradation-resistant biomaterials for expanded utility. This may allow clinical use in procedures generally reliant on ECM or synthetic materials in sheet formats, for instance as nerve conduits,^[46]^ or in tissues that demand increased mechanical resilience such treatment of tendinopathy.^[70–72]^

**Figure 6:**
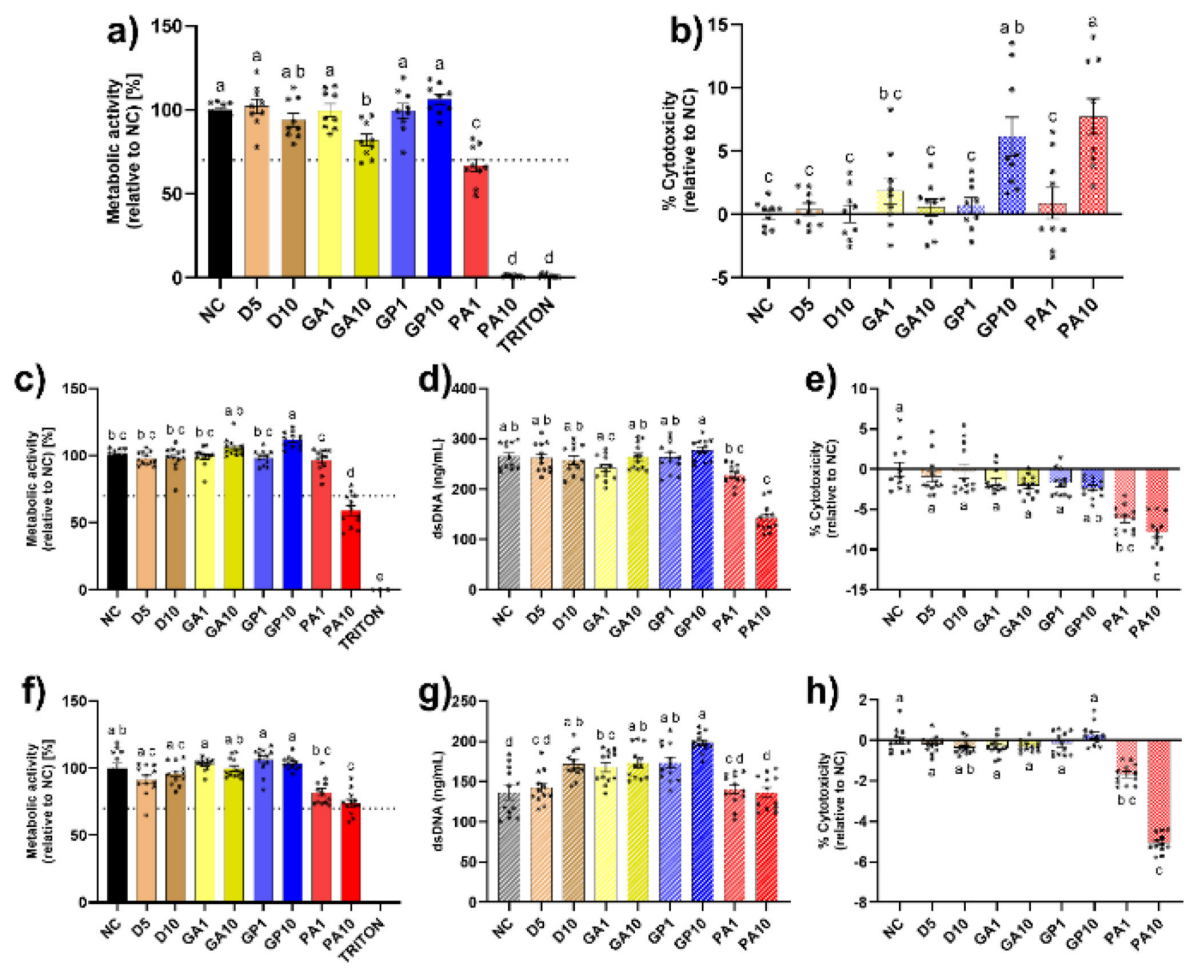
(a) Viability of SH-SY5Y cells as determined by metabolic activity following exposure to conditioned media, N=3, n=3; (b) Cytotoxicity effects of crosslinked gel eluant on SH-SY5Y cells, N=3, n=3; (c) Metabolic activity of L929 cells following elution based testing, with corresponding (d) dsDNA content and (e) LDH cytotoxicity values, N=2, n=6; (f) Effects of elution based testing on primary tenocyte metabolism with corresponding (g) dsDNA content and (h) LDH cytotoxicity assessment, N=2, n=6. Dotted line represents 70% cytotoxicity threshold. All data presented as mean ± SEM. Significance determined through Kruskal-Wallis non-parametric testing, with a threshold value of p<0.5, with shared lettering denoting statistically homogenous groupings.

Using the ISO 10993 framework designed for medical device testing, cytocompatibility was assessed through means of elution-based evaluation. This type of methodology examines labile chemicals that can leach from the sample in question. This was deemed suitable due to the proposed applications of ECM materials where samples are implanted *in vivo* and left to integrate into the damaged tissue, with continual exposure to bodily fluids facilitating elution of labile components. In SH-SY5Y testing, no apparent changes in metabolic activity were observed for GA1, and both GP samples in comparison to the non-crosslinked gels (Figure 6a). A significant decrease in metabolic activity was observed in GA10 samples, but this was above the 70% viability threshold outlined in ISO standards toxicity assessment. However, significant cytotoxicity was observed in both PA crosslinked hydrogels, with a seemingly complete cessation of metabolic activity following culture with PA10 conditioned media (Figure 6a). Despite this, direct placement of crosslinked gels onto a cell monolayer resulted in overall increases in metabolic activity across all sample types (Figure S8, Supporting Information), with remediation of PA1 cytotoxicity and near removal of cytotoxic effects from PA10 samples. Previous reports of the biocompatibility of PA reagent promote increased study as an alternative to GA,^[43, 73]^ however, effects of PA within this study contradict these findings. These cytotoxic effects were likely due to excess PA eluting from gels into the media solution, as evidenced by red colouration of the media following incubation (Figure S9, Supporting Information), which was not readily observed in PBS washes (Figure S3, Supporting Information), with variation in constituents and osmolarity potentially driving this increased elution. Differences between elution and direct testing were due to a dilution in this excess PA, as the same size gels were used for both testing types whilst the media volumes used in these tests differed, facilitating increased dilution in direct testing.

Using crosslinker doped media, cytotoxic effects on L929 cells were apparent for all GA concentrations examined (Figure S10, Supporting Information). Both GP and PA exhibited toxicity above 0.1 mM, however effects of PA were less pronounced with borderline toxicity for 0.5 mM, echoing results observed by Li *et al*.^[74]^ Declining toxicity through decreased concentration confirms the assumption that excess reagent is the probable cause of cytotoxicity observed in SH-SY5Y cells and discrepancy between elution and direct effects. Serial washing of gels may also account for the difference between apparent toxicity of GA and GP directly compared to their use in crosslinked gels, as observed by Nishiguchi and Taguchi,^[75]^ and more recently by Robinson *et al*.^[64]^

Working on the hypothesis that excess PA instigates cytotoxicity, crosslinking solutions were filtered using a 0.22 µm syringe filter to remove excess, non-solubilised PA prior to crosslinking. Use of this filtered reagent demonstrated no reduction in mechanical modulation effects (Figure S11, Supporting Information). Following crosslinking and media elution, media was notably less red than observed prior. Effects of this conditioned media on L929 were much different to those observed in SH-SY5Y testing, with only PA10 samples having a significantly reduced metabolism relative to NC gels, on average falling 11% under the 70% cytotoxicity threshold (Figure 6c). These metabolic effects were mirrored in quantity of dsDNA within the samples (Figure 6d), with significantly reduced dsDNA quantity in PA10 samples, indicating few cell numbers overall. This could indicate that the perceived impact on metabolic activity is due to less cells overall, and potentially not a cytotoxic effect. This was further evidenced by use of the LDH based cytotoxicity assay which demonstrated significant reductions in cytotoxicity for PA treated samples (Figure 6e), inferring reduced numbers of cell death.

For patellar tendon, shear moduli can range from 24 kPa to 121 kPa dependent on measured location and flexion of the tendon,^[76–78]^ with PA10 treated gels sitting within this range, therefore primary human tenocytes derived from patellar tendon were used for cytotoxicity evaluation. Significant reductions in tenocyte cell metabolism, relative to NC gels, were only observed from PA exposure, however these reductions were above the cytotoxicity threshold at 82% and 74% for PA1 and PA10 respectively (Figure 6f). All other crosslinked samples exhibited no significant change relative to non-crosslinked samples. Unlike L929 cells, there was no significant reduction in dsDNA content of the samples, indicating no large-scale difference in cell numbers (Figure 6g). There was a small yet significant decrease in the presence of LDH in PA treated samples, inferring a reduction in cytotoxicity (Figure 6h). Overall, this indicates that while tenocyte metabolism was challenged through PA exposure, a reduced number of cells appeared to have died compared to native controls. No significant changes in LDH derived cytotoxicity were observed for any other samples.

Despite multiple reports of GA toxicity,^[17, 21, 24]^ this was only observed when cells were directly exposed to GA reagent (Figure S10, Supporting Information), with no cytotoxicity detected from any GA crosslinked gel samples.

Further to cytotoxicity evaluation, preliminary qPCR was conducted on tenocytes grown on top of crosslinked hydrogels to investigate cell behaviour following direct contact. Growth on GP crosslinked gels demonstrated no significant changes for all assessed genes, whilst PA demonstrated no significant changes to pro-collagen expression, but decreased expression of matrixmetalloproteinase-1 (MMP1) and cartilage oligomeric matrix protein (COMP), common markers of tenocyte remodelling (Figure S12, Supporting Information).^[79]^ Growth of tenocytes on GA crosslinked gels resulted in significant decreases in pro-collagen expression.

## 3 Conclusions

In this study, we sought to understand how alternative crosslinking reagents (GP and PA) to glutaraldehyde (GA) can interact with and modulate bone derived ECM (bECM) hydrogels. A standardised crosslinking methodology was developed in this study, facilitating the direct comparison of three chemical crosslinkers for the first time in ECM hydrogels. Using this protocol, concentration dependencies were observed, and we developed a toolkit for finetuned modulation of bECM hydrogel properties. Both genipin (GP) and proanthocyanidins (PA) are fully capable of crosslinking bECM gels, with each reagent conferring differing properties. With GP crosslinking, low but significant boosts were made to gel mechanics; bECM hydrogels were strengthened without interference with degradation capabilities and GP cross-linked gels provided exemplary cytocompatibility. Use of PA resulted in the most drastic changes to bECM gels, with vastly increased mechanical strength, while resisting degradation and zero mineralisation. These effects however came with a detriment to cytocompatibility. Despite reported issues with GA crosslinking, negative consequences were only perceived in mineralisation testing within this study, whilst gels exhibited increased strengths, resisted degradation and retained cytocompatibility. Crosslinked bECM hydrogels have broader application prospects than non-treated hydrogel devices due predominantly to the increased hydrogel mechanical properties and stability.

## 4 Experimental Section

### 4.1 Materials and methods

A list of generic reagents and suppliers can be found in Table S3 (Supporting Information). Bovine tibiae were collected from 12-24 month-old animals sourced from an EU certified butcher (J. Broomhall Ltd, Dursley, Gloucestershire, UK).

### 4.2 Decellularization and ECM characterisation

Bone decellularization was performed according to an established protocol by Sawkins *et al*.^[80]^ In brief, bone slices were fragmented, and cancellous bone was demineralised in 25 mL g^-1^ of bone of 0.5 N HCl for 24 hours, before washing in five times volume of dH_2_O. Delipidation used a 1:1 mixture of chloroform:methanol at 30 mL g^-1^ of tissue for 1 hour, followed by washing in two times volume methanol then five times volume of PBS. Decellularization was achieved with 0.05% w/v trypsin and 0.02% EDTA at 30 mL g^-1^ of tissue at 37°C for 24 hours, then washed with PBS for another 24 hours to yield bone-ECM (bECM), prior to snap freezing and lyophilisation. All reaction steps were conducted under constant agitation.

#### 4.2.1 Quantification of DNA

Adapting the method of Gilbert *et al*.^[81]^ up to 100 mg of bECM was digested in 2 mL of lysis buffer (10mM Tris-HCl [pH 8], 100mM NaCl, 10mM EDTA [pH8], 0.5% w/v SDS, 5 µL/mL proteinase-K) and incubated at 50°C until complete solubilisation. An equal volume of phenol/chloroform/isoamyl alcohol (25:24:1) solution was added, mixed, then centrifuged at 10,000 rcf for 10 minutes at 4°C. The top aqueous phase was placed in a fresh tube with 3 M sodium acetate (pH 5.2) and ice cold 100% ethanol at double sample volume before freezing overnight at -80°C. Whilst frozen, samples were centrifuged at 10,000 rcf for 10 minutes at 4°C, collecting precipitated DNA at the bottom of the container. Supernatant was discarded and DNA was washed with 5 mL of 70% ethanol and centrifuged. Discarding the supernatant, the pellet was dried at room temperature then resuspended in 1 mL of 1x TE buffer and stored at -20°C until quantified using the Quant-iT Picogreen dsDNA assay kit, following the manufacturers protocol.

#### 4.2.2 Quantification of glycosaminoglycans

A 1 mL volume of PBE buffer solution (100 mM Na_2_HPO_4_, 10 mM EDTA, 10 mM L-cysteine, 125 µg mL^-1^ papain) was added to 10 mg of tissue and digested at 65°C until no tissue remained. Quantification was performed using a 1,9-dimethylmethylene blue (DMMB) colourimetric assay outlined by Zheng & Levenston.^[82]^ Briefly, DMMB was solubilised in 100% ethanol at 16 mg per 5 mL, before adding 40 mM glycine, 40 mM NaCl, dH2O and HCl to yield 1 L volume at pH1.5. To a 96-well plate, 20 µL of sample was added and mixed with 200 µL of DMMB solution. The plate was protected from light and read within 10 minutes on the Infinite M200 PRO UV plate reader (Tecan, Switzerland), with quantification based on the difference between absorbance at 525 nm and 595 nm. Samples were standardised against a stock solution of shark chondroitin sulphate.

### 4.3 ECM Hydrogel Generation

Following the method developed by Freytes *et al*.,^[7]^ a 10 mg mL^-1^ bECM digest was generated through solubilisation in a 0.01 M HCl solution containing 1 mg mL^-1^ pepsin for 48 hours at room temperature and constant stirring at 300 rpm. The bECM digest was adjusted to a final concentration of 8 mg mL^-1^ using a neutralisation buffer (one ninth volume 10xPBS, one tenth volume 0.1 M NaOH, remaining dilution volume of 1xPBS) with 200 µL of buffer for every 800 µL of digest. Hydrogel curing was conducted at 37°C for 40 minutes with gels used or processed immediately following gelation. All testing was conducted using 8 mg mL^-1^ bECM hydrogels, with an overview of gel sizes and volumes used for each experiment found in Table S4 (Supporting Information).

### 4.4 Crosslinking preparation & methodology

Stock crosslinking agents consisted of 50% w/v aqueous solution of GA (Sigma Aldrich, UK), purified genipin (Guangxi Shanyun Biochemical Science and Technology Co. Ltd, China), and grape seed extract (Bulk, UK) with a PA level of >95%. Due to solubility constraints of GP and PA, appropriate amounts of sample were weighed before solubilising in 100% DMSO. Dilution with PBS would yield final concentrations of 1, 10 and 50 mM crosslinkers in 5, 10 and 20% v/v DMSO, respectively. Aqueous GA was diluted with PBS with appropriate volumes of DMSO added as a vehicle control.

A standardised approach to crosslinking was developed during this project, using solutions at the desired concentration (1, 10 and 50 mM) added to bECM hydrogels at three times volume of bECM hydrogel. Samples were placed on an orbital shaker (Digital Mini Rotators, Thermo Scientific, UK) at 150 rpm for 2 hours, before disposing of the crosslinking solution, rinsing the gel with equal volume of PBS and then washing four times with PBS in 30-minute intervals. Investigations on a range of crosslinking times was initially conducted. A cross-linking time of 2 hours provided observable differences in gel robustness and crosslinker concentration dependent effects were noted and therefore carried forward for characterisation. Where possible, samples were tested or further processed immediately following crosslinking, alternatively samples were stored in PBS solution at 4°C overnight prior to further processing or testing.

### 4.5 Crosslinked bECM hydrogel characterisation

The characterisation methodologies utilised in this work encompass a broad range of tests designed to interrogate the physicochemical and mechanical behaviours of bECM hydrogels and the effects of externally mediated crosslinking. These methods have been chosen with reference to the interdisciplinary framework generated by Martin-Saldaña *et al*. ^[83]^ to provide more generalised and comparative datasets for comparisons with future works.

#### 4.5.1 ATR-FTIR

Crosslinked bECM hydrogels were frozen overnight at -80°C and lyophilised (ScanVac CoolSafe, Labogene, Denmark). Dried samples were kept whole and analysed on the Agilent Cary 630 FTIR spectrophotometer with the attenuated total reflectance (ATR) module. Absorbance was measured between 4000 and 750 cm^-1^ wavelengths at an 8 cm^-1^ resolution for 32 scans. Spectra were presented using OriginPro 2023b software, normalised through with manual baseline corrections.

#### 4.5.2 Electron microscopy

Samples were prepared using the Leica EM ICE high pressure freezer (HPF) (Leica, Austria). Central sections of gel samples were removed with a 3 mm tissue punch and placed into tissue planchettes. Using another planchette, the sample was covered and inserted into the HPF and frozen under a pressure of 2100 bar. Following freezing, samples were decanted into a liquid nitrogen bath where planchettes were separated and the sample was transferred into a pre-cooled freeze dryer (Lablyo Mini, Frozen in Time Ltd, UK) for drying under vacuum at -50°C. Samples were attached to aluminium stubs and platinum coated for 45 seconds at a current of 30 mA (Agar Scientific Automatic Sputter Coater, Agar Scientific, UK), yielding a 15 nm thick platinum deposition. Following coating, samples were imaged using a Zeiss Crossbeam 550 SEM (Zeiss, Germany) in high vacuum at an accelerating voltage of 2 kV.

Using Fiji ImageJ,^[84]^ fibre diameter and porosity were assessed using three to four images per condition. Images were enhanced through brightness and contrast adjustment prior to analysis. Fibre diameter was performed using the ‘Ridge detection’ plugin tool (line width = 3.5, high contrast = 100, low contrast = 50, sigma = 2.50, lower threshold = 1, upper threshold = 3). For pore analysis, images were threshold adjusted and the ‘Analyze Particles’ tool was used. To assess network connection density, threshold adjusted images were skeletonized and connections counted using the ‘Analyze skeleton’ tool. Example images of analysis workflows can be found in Figure S13 (Supporting Information).

#### 4.5.3 Rheology

Hydrogels of a 1.3 mL volume were cured in a 22 mm circular mould with rheology performed immediately following crosslinking. Testing was conducted using the Physica MCR 302 rheometer (Anton Paar, Germany), with a 25 mm smooth parallel plate geometry. Gels were placed onto a preheated stage at 37°C and compressed until a normal force of 0.1-0.5 N was registered. An amplitude sweep was performed from 0.01-200% strain at a constant angular frequency of 1 rad/second for measurement of storage and loss moduli including material deformation, with a frequency sweep performed at a constant 0.5% strain and range of 0.01-200 rad/second angular frequency to assess mechanical moduli within the non-destructive range. Data capture and analysis was performed through the RheoCompass software.

#### 4.5.4 Swelling degree

Freeze dried, crosslinked gels were weighed to the nearest 0.1 mg then immersed in a PBS solution with 1% AB/AM, and incubated at 37°C. At designated time points gels were removed from solution, gently blotted with filter paper and weighed to the nearest 0.1 mg. The swelling degree was expressed as the percentage change in weight from original dry weight using equation [1].

[1] $$\frac{\left(W_{wet}-W_{dry\;}\right)}{W_{dry\;}}\times 100=\text{\%}\;swelling\;degree\;$$

Following swelling conclusion, samples were freeze dried and weighed to the nearest 0.1 mg for comparison against starting dry weights, allowing elucidation of sample weight loss through hydrolysis.

#### 4.5.5 Collagenase degradation

Initial assessments on 10 and 50 mM crosslinked samples were conducted with hydrogels immersed in four times gel volume of Hanks Balanced Salt Solution (HBSS) (with 1.26 mM CaCl_2_, 0.5 mM MgCl_2_ and 1% AB/AM) with addition of 100 collagen degrading units (CDU) mL^-1^ of collagenase type-I for tested samples. Samples were placed on an orbital plate shaker at a constant 250 rpm at 37°C. At designated time points, the full volume of solution was removed and replaced with fresh solution. Samples were immediately stored at -20°C until all time points were taken and analysed using the ninhydrin assay.

Degradation assessment of 1 and 10 mM crosslinked samples was conducted following the protocol outlined by Výborný *et al*.^[65]^ The bECM hydrogels were crosslinked then immersed in 150 CDU collagenase solution, alongside non-crosslinked control samples. Following complete dissolution of non-crosslinked gels, samples were removed from solution, rinsed with deionized water and lyophilised overnight before weighing. Dry weights of crosslinked gels were expressed as percentage of remaining weight against a control gel in HBSS only using the formula:

$$\frac{W_{digested\;gel\;}}{W_{control\;gel\;}}\times 100=\text{\%}\;remaining\;weight\;$$

#### 4.5.6 Ninhydrin assay

A protocol was optimised for a 96-well microplate format. A 2% w/v ninhydrin solution was created using 2 g of ninhydrin (Acros Organics, UK) in 95 mL isopropanol and 5 mL of 10% v/v glacial acetic acid in dH_2_O, alongside a glycine standard curve in the range of 1.95-500 µg mL^-1^ (26 µM to 6.6 mM). To 50 µL of sample, an equal volume of ninhydrin solution was added and reacted at 37°C for 1 hour. The sample was then diluted with 200 µL of ice-cold isopropanol to quench the reaction and absorbance was read at 570 nm.

#### 4.5.7 Mineralisation assessment

Crosslinked bECM hydrogels were immersed in SBF which was prepared according to the method outlined by Kokubo and Takadama.^[85]^ Briefly, SBF solution consisted of NaCl, NaHCO_3_, KCl, K_2_HPO_4_·3H_2_O, MgCl_2_·6H_2_O, CaCl_2_, Na_2_SO_4_ dissolved in ultrapure water and buffered with Tris base and 1 M hydrochloric acid to a pH of 7.4.^[85]^ Crosslinked hydrogels were fully immersed in excess volume of SBF solution and incubated at 37°C for 28 days, with samples inverted twice daily. Following incubation, samples were visually assessed for calcification and alizarin red staining. For alizarin staining, samples were immersed in a 1% w/v alizarin red solution in water for 30 minutes, then washed thoroughly in deionized water until no unbound dye remained prior to imaging.

### 4.6 Cytocompatibility assessment

Crosslinked hydrogels compatibility was comprehensively tested utilising three different cell types: SH-SY5Y, L929 and primary human tenocytes. Due to previously published work by the group,^[46]^ SH-SY5Y neuroblastoma cells (ATCC CRL-2266™, ATCC, US) were selected for a neurotoxicity model.^[86]^ The L929 mouse fibroblast cell line (ATCC CCL-1™, ATCC, US) is a commonly used cell line for cytotoxicity assessments. Primary human tenocytes (P10968, Innoprot, Spain) were used for compatibility assessment to evaluate effects of crosslinked bECM hydrogels for expanded use on mechanically resilient tissues.

#### 4.6.1 Tissue culture

The SH-SY5Y cells were cultured in a 1:1 mix of EMEM and Ham’s F-12 nutrient mixture, with 10% FBS, 1% v/v AB/AM and 1% non-essential amino acids (NEAA). Culture medium was replaced every 4-5 days and incubated within standard culture conditions. Cells were passaged at 80-90% confluency and a split ratio of 1:10 as recommended by the ATCC. Cells were used from passage 15 to 20.

The L929 cells were cultured in Eagles modified MEM (EMEM) supplemented with 10% v/v foetal bovine serum (FBS), 1% v/v antibiotics and antimycotics (AB/AM) (penicillin [100 units mL^-1^], streptomycin [100 µg mL^-1^], amphotericin B [0.25 µg mL^-1^]) and 50 µg ml^-1^ of gentamicin (Gibco, UK) under standard culture conditions (37°C, 95% air and 5% CO_2_). Medium was replaced every 3 days with cells passaged using trypsin/EDTA at 80-90% confluency using a split ratio between 1:5 and 1:8 as recommended by ATCC. Cells were used between passage 11 and 13.

Tenocytes were cultured in the manufacturer’s tenocyte growth media (P60177, Innoprot, Spain) with the provided supplementation and 50 µg mL^-1^ gentamicin, under standard culture conditions (37°C, 95% air and 5% CO_2_) and medium replacement every 2-3 days. As recommended by the supplier, cells were cultured at a minimum density of 7,500 cells cm^-2^. Cells were used within the guaranteed 10 population doublings.

#### 4.6.2 Cytotoxicity Evaluation

Testing was conducted in reference to the ISO 10993 framework, following guidelines outlined in part 5 and part 12.^[87–88]^ Elution based extract testing was primarily conducted using complete media conditioned by 24-hour exposure to crosslinked hydrogels at 37°C under constant agitation. Volume of media used was calculated using a sample surface area/elution volume extraction ratio outlined in ISO 10993-12 framework of 3 cm^2^ per 1 mL of solution. Direct contact testing was conducted through sample placement on top of the cell monolayer, covering approximately 10% of the growing surface area.

#### 4.6.3 Cell viability, cytotoxicity and proliferation

Cells were cultured in 96 well plates at a density of 5000 cells per well, equating to approximately 15,000 cells cm^-2^ growing surface. Cells were incubated for 24 hours to allow for cell attachment and development of a sub-confluent monolayer prior to addition of conditioned media. Cells were incubated with conditioned eluant for 24 hours then assessed for response. Cell viability was evaluated using PrestoBlue™ reagent diluted to 10% v/v concentration in complete culture media, then added to cells and incubated for 2 hours. The media was then transferred to a black 96-well plate and fluorescence was read at 560/590 nm ex/em.

To estimate cell number changes double-stranded DNA content was assessed through Quant-iT Picogreen dsDNA assay kit. Cells were gently washed twice with PBS to remove excess media and PrestoBlue™, then subjected to one freeze thaw cycle in TE buffer to lyse cells. Samples were transferred to a black 96-well plate and equal volume of PicoGreen working reagent was added. Plates were incubated for 5 minutes before reading fluorescence at 480/520 nm ex/em. Fluorescent readings were normalised against a standard curve of λDNA provided within the assay kit.

The CyQuant™ LDH cytotoxicity assay was conducted using media from the tested cultures where 50 µL was combined with 50 µL of reaction buffer and kept protected from light for 30 minutes. Following incubation, 50 µL of stop solution was added and the samples read for absorbance at 490 nm with a reference wavelength of 680 nm.

#### 4.6.4 qPCR analysis

Hydrogels of 50 uL volume were formed on top of glass cover slips then crosslinked. Following the crosslinking and washing process, coverslips were placed into tissue culture plates and washed with culture media prior to tenocyte seeding with a density of 1×10^4^ cells cm^-2^ growing surface. Following three days of culture cells were lysed and RNA isolated using a Qiagen RNeasy kit. Assessed genes were pro-collagen 1 subunit A1 (COL1A1), pro-collagen 3 subunit A1 (COL3A1), matrix metalloproteinase 1 (MMP1), cartilage oligomeric matrix protein (COMP), and interleukin 6 (IL-6). Gene expression was normalised to expression of tenocytes grown on base tissue culture plastic.

### 4.7 Data presentation and statistical analyses

Graphical representations and statistical analysis were performed through GraphPad Prism 10 (GraphPad Software LLC, Boston, USA). Experiments were performed on sample replicates with ‘N’ representing the number of experimental repeats, and ‘n’ denoting the number of gel replicates tested within an experiment. Data is presented as mean ± standard error of mean. Statistical testing was conducted according to number of samples and variables present and tested for normality prior to assessment. Normally distributed data was assessed through either one-way ANOVA (multiple comparisons, one variable) or two-way ANOVA (multiple comparisons, multiple variables), with post-hoc testing using Tukey’s correction. Any heteroscedasticity present within sampling was accounted for through Log(Y) transformation of data to maintain valid ANOVA assumptions. Non-normally distributed data was tested using a Kruskal-Wallis ANOVA test with Dunn’s post-hoc multiple comparisons test. Significance was determined as p<0.5 and is displayed through compact letter displays, where shared letters denote statistically homogenous (non-significant) groups.

## Supplementary Material

Supplementary information

## Figures and Tables

**Table 1:** Overview of rheological parameters captured and explored.

Measurement			Definition	Reason Recorded	Equation
**Complex Modulus**	G*	Pascals (Pa)	Overall resistance to shear deformation.	Direct measure of structural rigidity when tested below yield stresses.	G^*^ = *G*′ + *iG*″
**Storage Modulus**	G′	Pascals (Pa)	Measure of stored energy during deformation.	Material rigidity attributed to the solid- like proponent of the hydrogel.	$G^{\prime }=\frac{\sigma_{0}}{e_{0}}\cos\delta$
**Loss Modulus**	*G″*	Pascals (Pa)	Measure of dissipated energy during deformation.	Proportion of rigidity that comes from the viscous flow.	$G^{\prime \prime }=\frac{\sigma_{0}}{e_{0}}\sin\delta$
**Damping** **Factor**	Tan δ	Unitless	Ratio of the viscous and elastic moduli.	Illustrates dominance of elastic of viscous behaviours in the material.	$Tan\;\delta =\frac{G^{\prime \prime }}{G^{\prime }}$
**Linear Viscoelastic Region**	LVE-R		Linear range present before irreversible structural deformation.	Identifies maximum level of strain before onset of structural breakdown.	
**Linearity Limit**	γL	Percent (%)	End-point strain value before irreversible deformation.	Provides a single value to compare onset of structural damage.	
**Yield Stress**	T_y_	Pascals (Pa)	Shear stress value at onset of plastic deformation.	As above.	$\tau =\frac{F}{A}$
**Crossover** **Strain**		Percent (%)	Strain value where G′=G″.	Provides a single value for comparison of complete structural breakdown.	
**Flow Point**	T_f_	Pascals (Pa)	Shear stress needed begin material flow.	As above.	$\tau =\frac{F}{A}$
**Flow Transition Index**		Unitless	The ratio between flow stress and yield stress.	Evaluates the transition breakdown behaviour of the material; brittle or soft.	$\;F.T.I.\;=\frac{\tau_{f}}{\tau_{y}}$
