# Supplementary material for: Comparison of crosslinking reagents and their impacts on bone derived ECM hydrogels
Source: Adv Healthc Mater. Author manuscript; Available in PMC 2026 Apr 16. (PMC7619007; doi:10.1002/adhm.202503252)
Supplement: Supplementary information [file EMS213029-supplement-Supplementary_information.pdf]

Comparison of crosslinking reagents and their impacts on bone derived ECM hydrogels

Joshua N. Jones, I-Ning Lee, Andreas Rialas, Simon C. Kellaway, Rabea Loczenski, Christopher Parmenter, and Lisa J. White*

J.N. Jones, I. -N. Lee, A. Rialas, S.C. Kellaway, R. Loczenski, C. Parmenter, L.J. White – School of Pharmacy, University of Nottingham, Nottingham, NG7 2RD, UK.

J.N. Jones, I. -N. Lee, L.J. White – Biodiscovery Institute, University of Nottingham, Nottingham, NG7 2RD, UK.

C. Parmenter – Nanoscale and Microscale Research Centre (nmRC), University of Nottingham, Nottingham, NG7 2RD, UK.

S.C. Kellaway – Centre for Nerve Engineering, University College London, London UK. Affiliation at time.

S.C. Kellaway – Department of Pharmacology, UCL School of Pharmacy, University College London, London, WC1N 1AX, UK. Affiliation at time.

*Corresponding author: lisa.white@nottingham.ac.uk

Orcid IDs: J.N. Jones https://orcid.org/0000-0003-3926-345X ; I. -N. Lee https://orcid.org/0000-0001-8672-8104 ; S.C. Kellaway https://orcid.org/0000-0001-5262-792X ; C. Parmenter https://orcid.org/0000-0002-2092-0555 ; L.J. White https://orcid.org/0000-0003-0571-999X

Supplementary Information

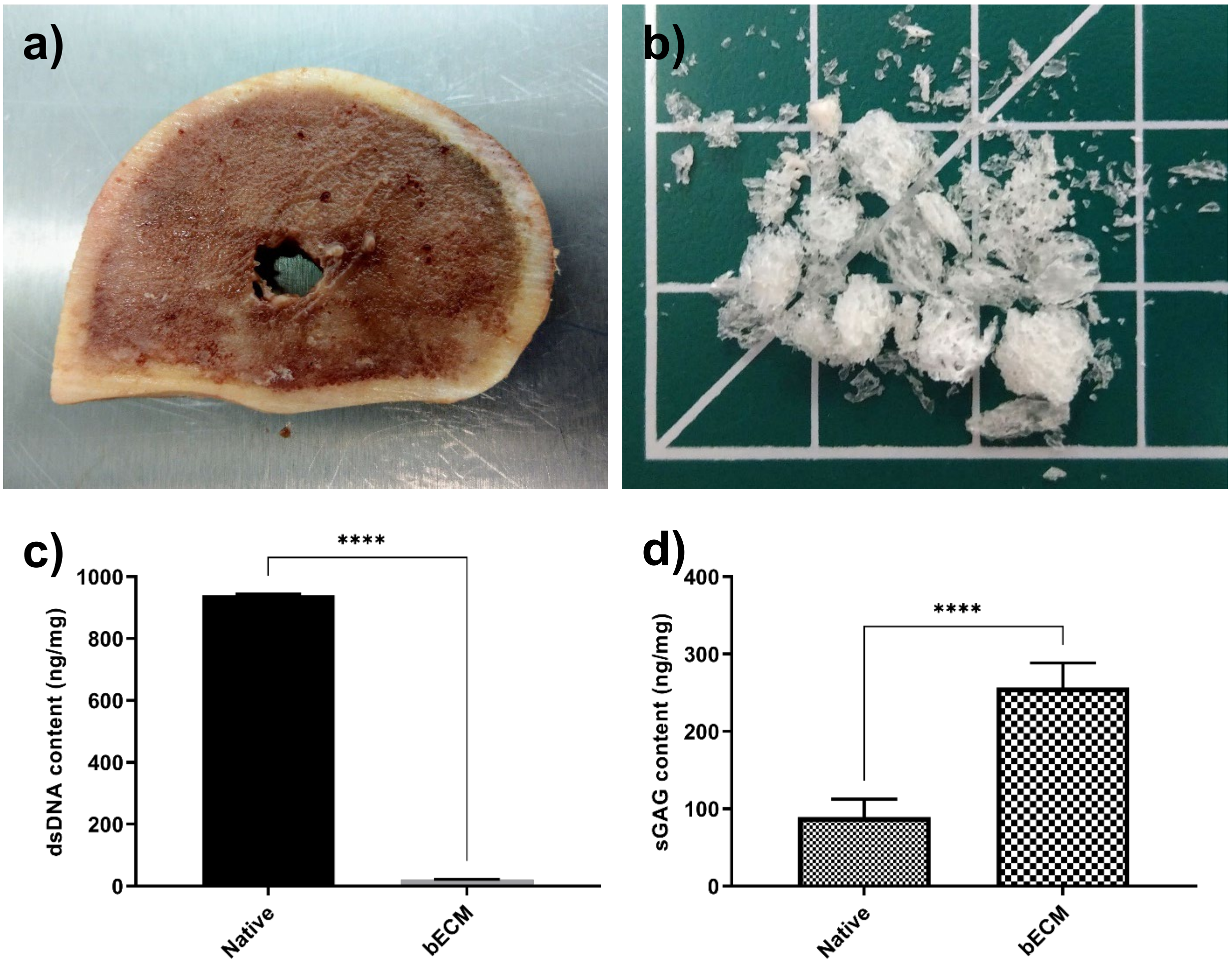

Figure S1: The decellularization process of fresh bone tissue (a) yielded a fragmented, porous bECM scaffold (b). Decellularisation was confirmed through dsDNA quantification (c) and sGAG quantification (d). Significance determined through t-test, with **** denoting p<0.0001.

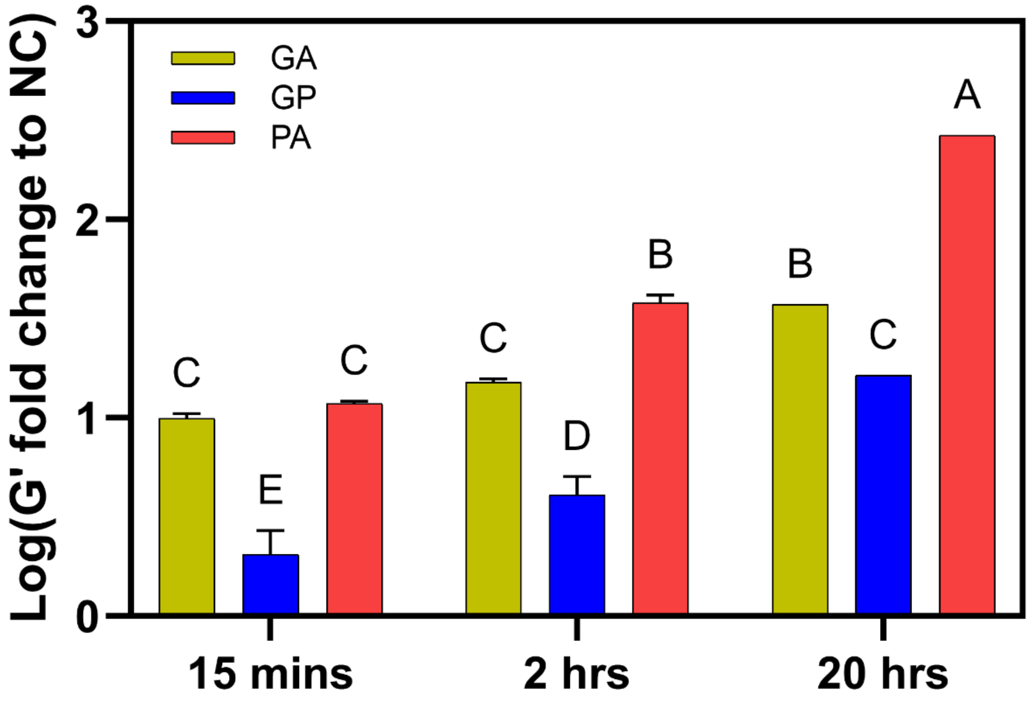

Figure S2: Preliminary assessment of bECM hydrogel storage moduli (G') crosslinked over differing time periods. Differences were observed within 15 minutes, however greater differences were seen over increased time, with significant differences observed between all three reagents after 2 hours. Data represented as log(y) transformation of n=3 (15 mins and 2 hrs) and n=1 (20 hrs). Significance determined through two-way ANOVA and Tukey post-hoc test, with shared lettering denoting no significant difference ($p>0.5$).

Table S1: Crosslinker molarity concentrations used and their equivalent weight per volume concentrations. A 20% volume of DMSO was used for original 10- and 50-mM concentrations during crosslinking optimisation.

**Crosslinker**	**Mw**	**Concentration**			
		Molarity (mM)	% w/v	Weight (mg/mL)	DMSO %v/v
**Glutaraldehyde**	100.12	1	0.01	0.1	5
		10	0.1	1	10, 20
		50	0.5	5	20
**Genipin**	226.23	1	0.023	0.23	5
		10	0.226	2.26	10, 20
		50	1.113	11.31	20
**Proanthocyanidins**	592.5	1	0.059	0.59	5
		10	0.592	5.92	10, 20
		50	2.963	29.63	20

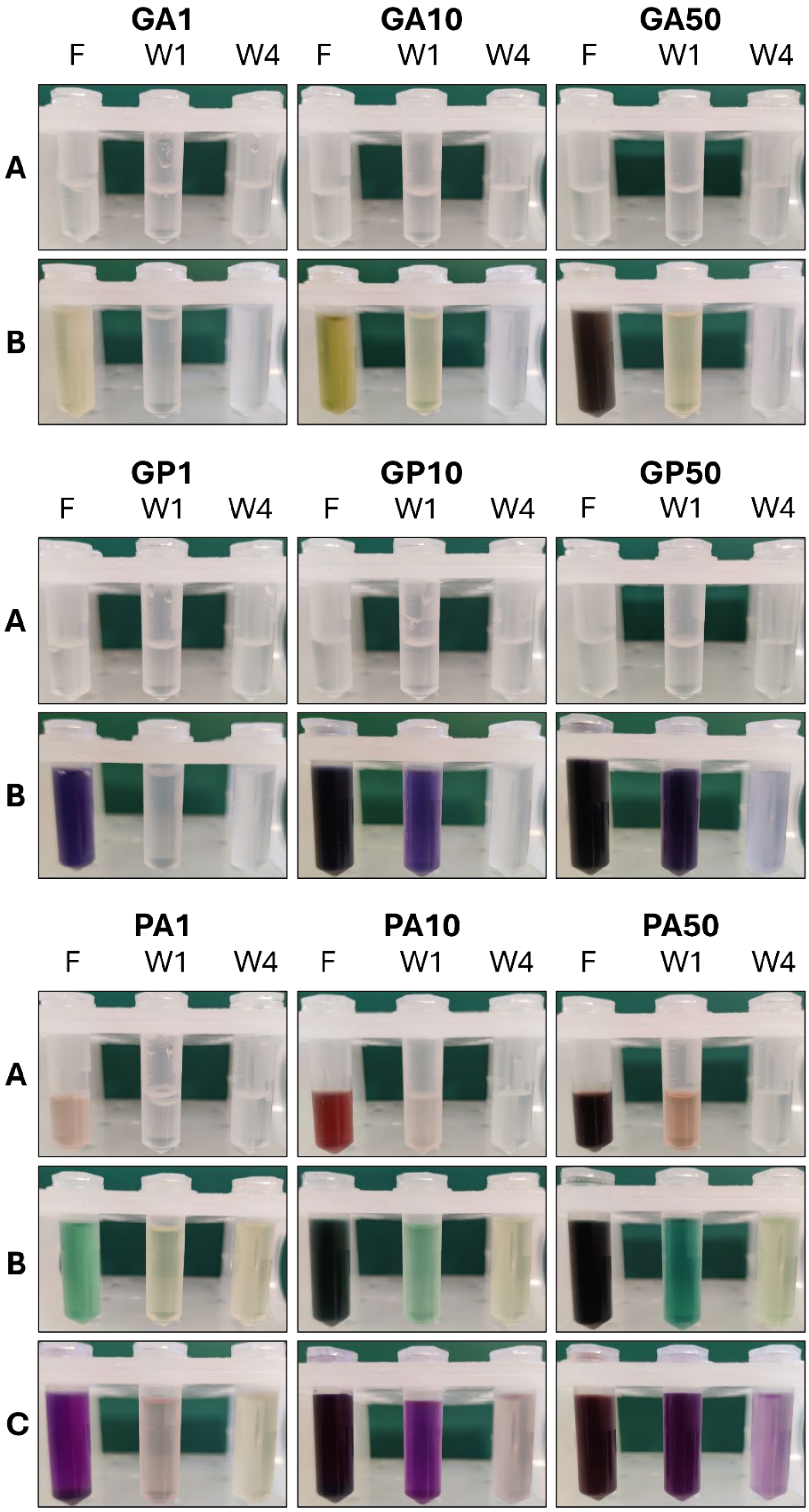

Figure S3: Fresh crosslinking reagents alongside PBS washes following gel treatment were collected (A) and assessed for residual crosslinking agent through addition of saturated glycine solution for GA and GP, and 0.1% DMAC for PA (B). Yellow colouring in PBS wash 1 [W1] in GA samples indicated presence of residual GA, however its increased solubility resulted in seemingly complete removal by wash 4 [W4]. However, both GP and PA reagents demonstrated retention of crosslinking agents following the four wash steps particularly at 50 mM concentrations, indicated by the blue colouring for GP reacted with glycine, and the blue/green colouring of PA and DMAC. Further reaction and degradation between PA and DMAC resulted in purple colouration (C), illustrating residual PA present within ≥10 mM concentrations.

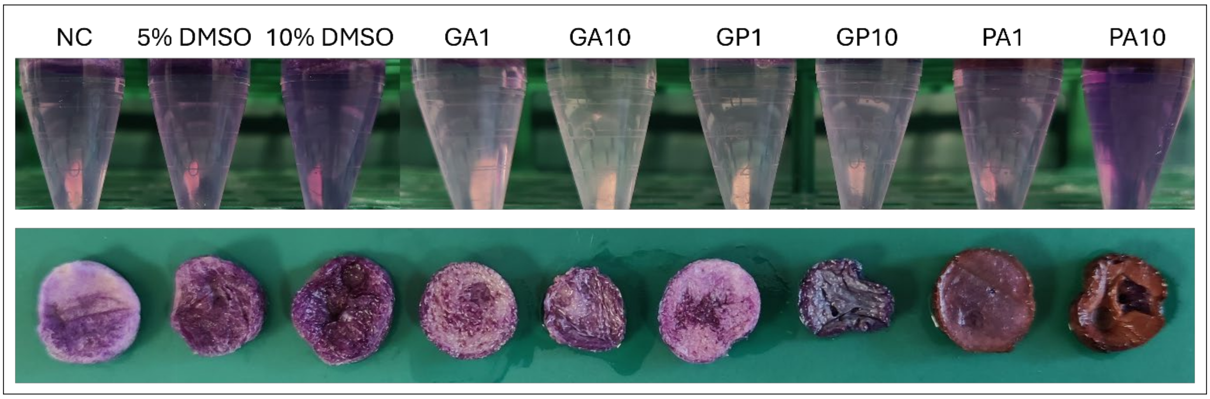

Figure S4: Representative images of crosslinked gels following interaction with ninhydrin solution.

Table S2: Calculated values of network connections in SEM images of crosslinked bECM hydrogels. Images analysed using imageJ.

Crosslinker	Network Junctions	
	**Total number**	**Per µm^2**
NC	4855 ± 597	50 ± 6
D5	4982 ± 390	51 ± 4
D10	5461 ± 326	56 ± 3
GA1	5513 ± 1569	56 ± 16
GA10	6557 ±923	67 ± 9
GP1	4822 ± 139	49 ± 1
GP10	6301 ± 853	64 ± 9
PA1	4407 ± 655	45 ± 7
PA10	6594 ± 300	67 ± 3

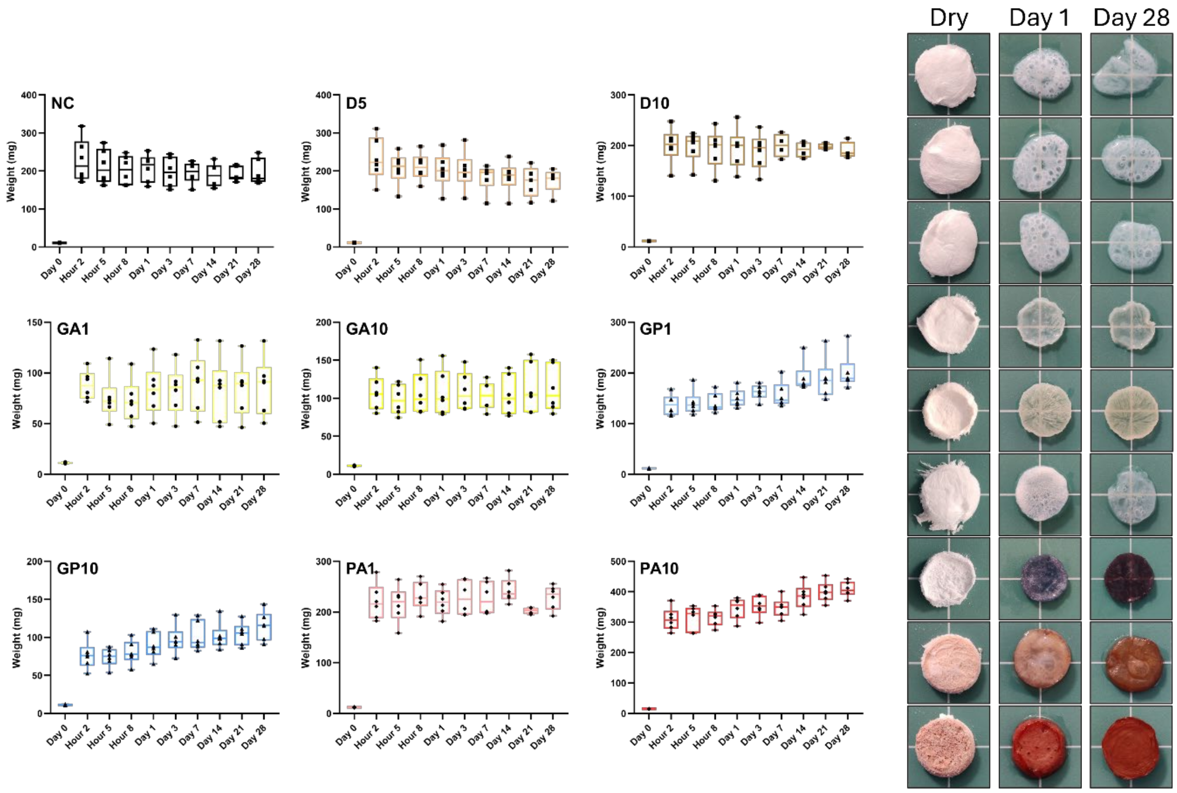

Figure S5: Boxplots of gel swelling over time with representative images of dried gels and rehydrated gels at day1 and day 28 (images not to scale). From top to bottom, images are NC, D5, D10, GA1, GA10, GP1, GP10, PA1, and PA10.

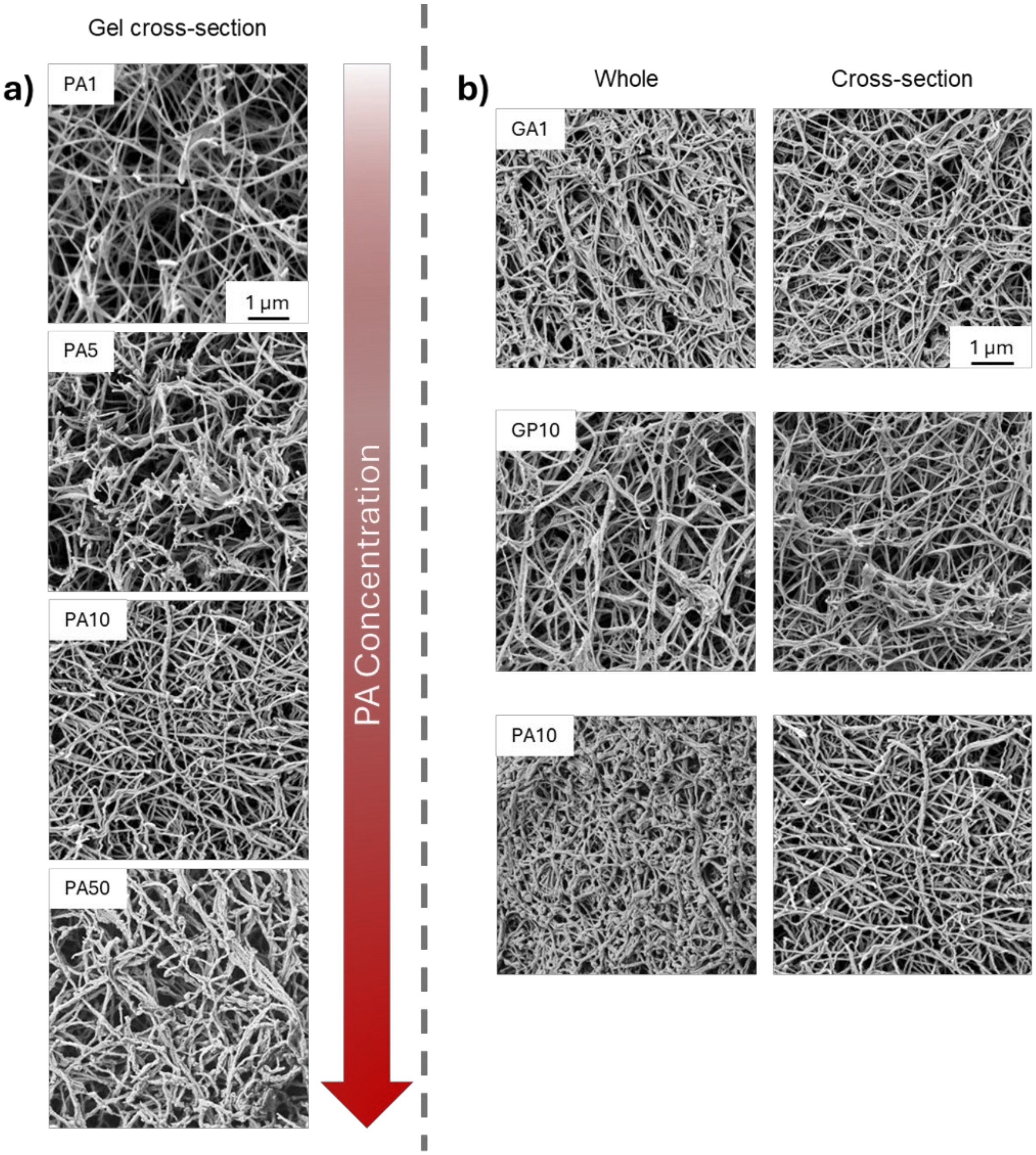

Figure S6: (a) Representative images of bECM hydrogels crosslinked with increasing concentrations of PA crosslinker; (b) Representative images of the surface and internal structures of crosslinked bECM hydrogels. Scale bars apply to all images.

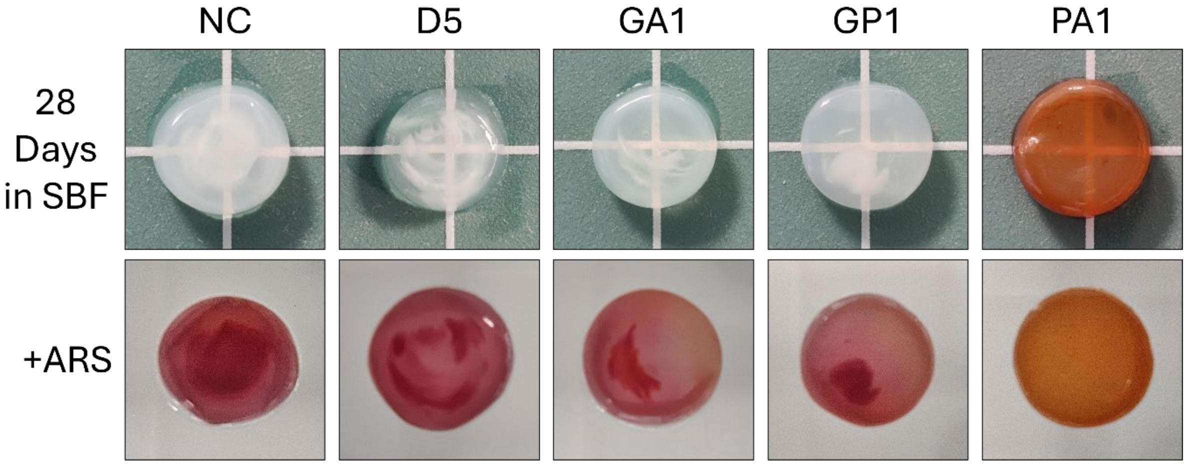

Figure S7: Representative images of alizarin red stained (ARS) crosslinked hydrogels following mineralisation assessment. Images not to scale.

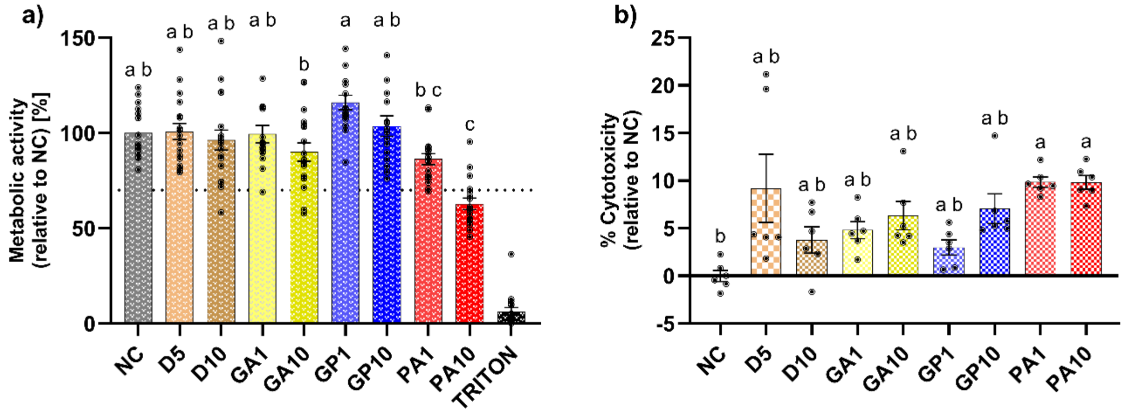

Figure S8: Metabolic activity of SH-SY5Y cells in direct contact with crosslinked gels (Left); LDH cytotoxicity assessment of cells in direct contact with crosslinked gels (Right). N=2, n=3, shared lettering denotes statistical homogeneity.

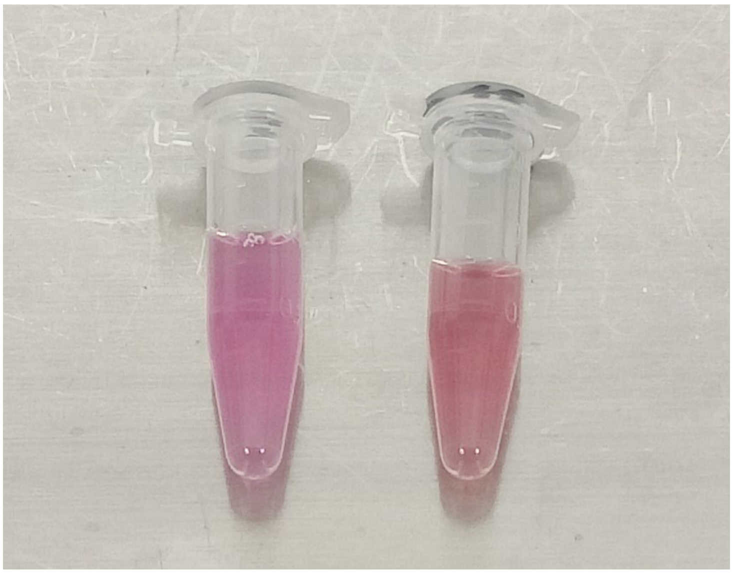

Figure S9: Leaching of proanthocyanidins into cell culture media can be seen where plain media (Left) takes on red colouration (Right).

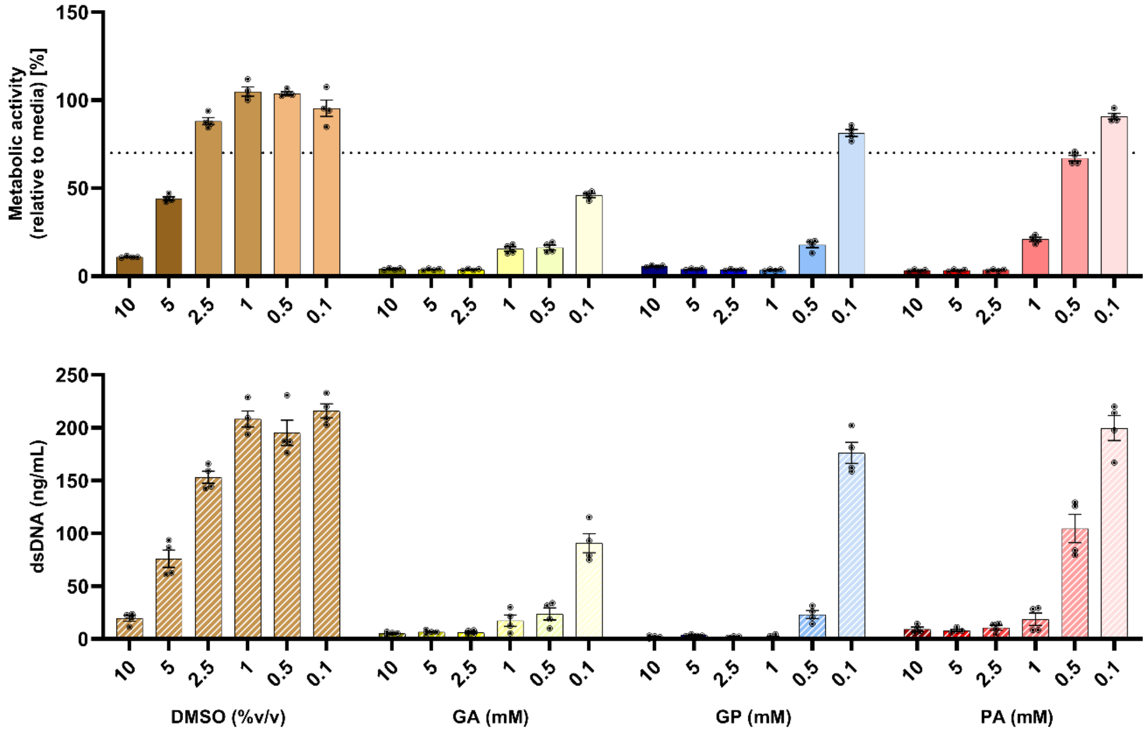

Figure S10: Metabolic activity of L929 cells following 24 hours culture in crosslinker containing media (Top); Quantification of dsDNA content of cell monolayer following culture in crosslinker doped media (Bottom). N=2, n=2.

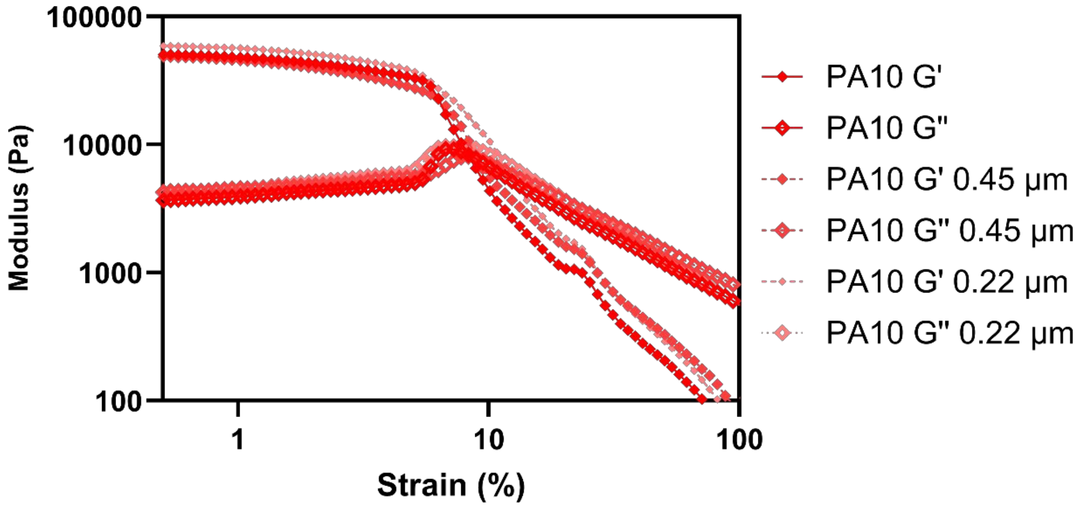

Figure S11: Amplitude sweep profiles of crosslinked bECM gels using unfiltered and filtered PA solution.

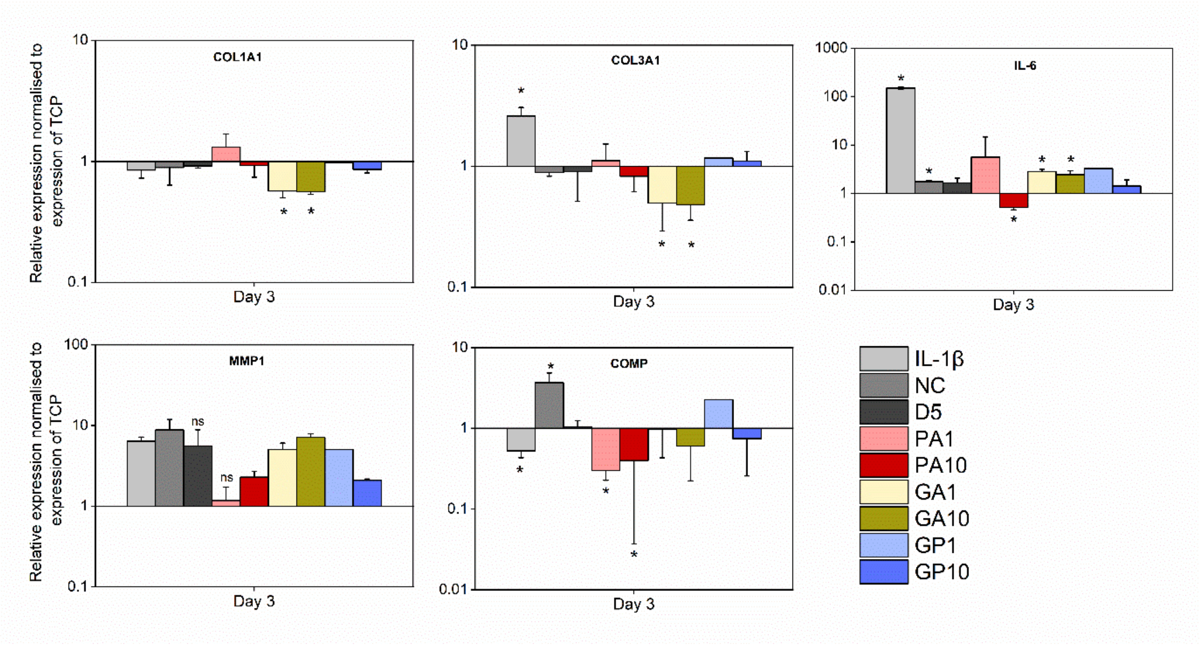

Figure S12: Preliminary qPCR assessment of primary tenocytes grown on crosslinked hydrogels. Significance determined through one way ANOVA of n=3, with * denoting $p<0.05$.

Table S3: List of material suppliers

Materials or Chemical	Supplier & Product code
1,9-dimethylmethylene blue	Sigma Aldrich, 341088, UK
Acetic acid (glacial)	Fisher Scientific, A/0400/PB17
Antibiotic-Antimycotic solution (AB/AM)	Sigma Aldrich, A5955-100ML, UK
Brass compression olive rings (15 & 22 mm)	B&Q, UK
Centrifuge tubes	Greiner Bio One, UK<br>15 mL – 188261-N<br>50 mL – 210270
Chloroform	Fisher Scientific, C/4960/PB17
Chondroitin sulphate	Sigma Aldrich, C4384, UK
Collagenase	Sigma Aldrich, C6885-100MG, UK
CyQuant LDH cytoxicity assay kit	Invitrogen, C20301, UK
Disodium phosphate	Sigma Aldrich, 71649, UK
Eagle's Minimum Essential Media (EMEM)	Sigma Aldrich, M4655, UK
Ethanol	Fisher Scientific, E/0650DF/17, UK
Ethylenediaminetetraacetic acid (EDTA)	Fischer Scientific, D/0700/53, UK
F-12 nutrient mix	Gibco, 21765-029, UK
Genipin	Guangxi Shanyun Biochemical Science and Technology Co. Ltd, China
Gentamicin	Sigma Aldrich, G1397, UK
Glutaraldehyde	Sigma Aldrich, 340855, UK
Glycine	Sigma Aldrich, G6600, UK
Grape seed extract (>95% PA)	Bulkpowders, Sports Supplement Ltd, UK
Hanks balanced salt solution (HBSS)	Cytiva, SH30268.01, USA
Hydrochloric acid	Sigma Aldrich, 435570, UK
L-cysteine	Sigma Aldrich, C1276-50G, UK
Methanol	Fisher Scientific, M/4000/17
Ninhydrin	Acros Organics, 415720100, UK
Non-essential amino acids	Gibco, 11140-050, UK
Papain	Sigma Aldrich, P3375-25G, UK

Penicillin and Streptomycin (P/S)	Fisher Scientific, 11528876, UK
Phenol/Chloroform/Isoamyl (25:24:1)	Sigma Aldrich, 77617, UK
Phosphate buffered saline (PBS)	Fisher Scientific, 1282-1680, UK
Quant-iT Pico green assay kit	Life technologies, P11496, UK
Prestoblue® reagent	Invitrogen, A13262, UK
Proteinase K	Invitrogen, 25530-049, UK
RNeasy kit	Qiagen, 74104, UK
Sodium chloride (NaCl)	Fisher Scientific, S/3160/63, UK
Sodium dodecyl sulphate (SDS)	Sigma Aldrich, L3771-500G, UK
Sodium hydroxide (NaOH)	Sigma Aldrich, S8045, UK
Tissue culture flasks, 75cm^2	Thermo Scientific, 156499, UK
Tris-EDTA (TE) buffer	PanReac AppliChem, 7I012281, UK
Tris-HCL	Fisher Scientific, 77-86-1, USA
Trypsin	Thermo Fisher Scientific, 15090-046, UK
Well plates	Corning, USA<br>6 well – 351146<br>12 well – 351143, 353043<br>24 well – 351147<br>48 well – 351178<br>96 well – 353072, 353072

Table S4: Size and volumes of gel and crosslinking agents used for each experiment

Experiment	Mold size	Gel volume (µL)	Crosslinker volume (mL)	Wash reagent	Gel state
**FTIR**	15 mm	750	2.5	PBS	Freeze-dried
**SEM**	8 mm	150	0.5	PBS + Ultrapure water	HPF & dried
**Rheology**	22 mm	1500	4.5	PBS	Fresh
**Swelling**	15 mm	750	2.5	PBS	Freeze-dried
**Degradation**	8 mm	150	0.5	PBS	Fresh
**Mineralisation**	8 mm	150	0.5	PBS	Fresh
**Elution cytotoxicity**	8 mm	200	1	PBS + AB/AM	Fresh
**Direct cytotoxicity**	8 mm	150	0.5	PBS + AB/AM	Fresh

PBS – phosphate buffered saline, HPF – high pressure frozen, AB/AM - Antibiotic-Antimycotic

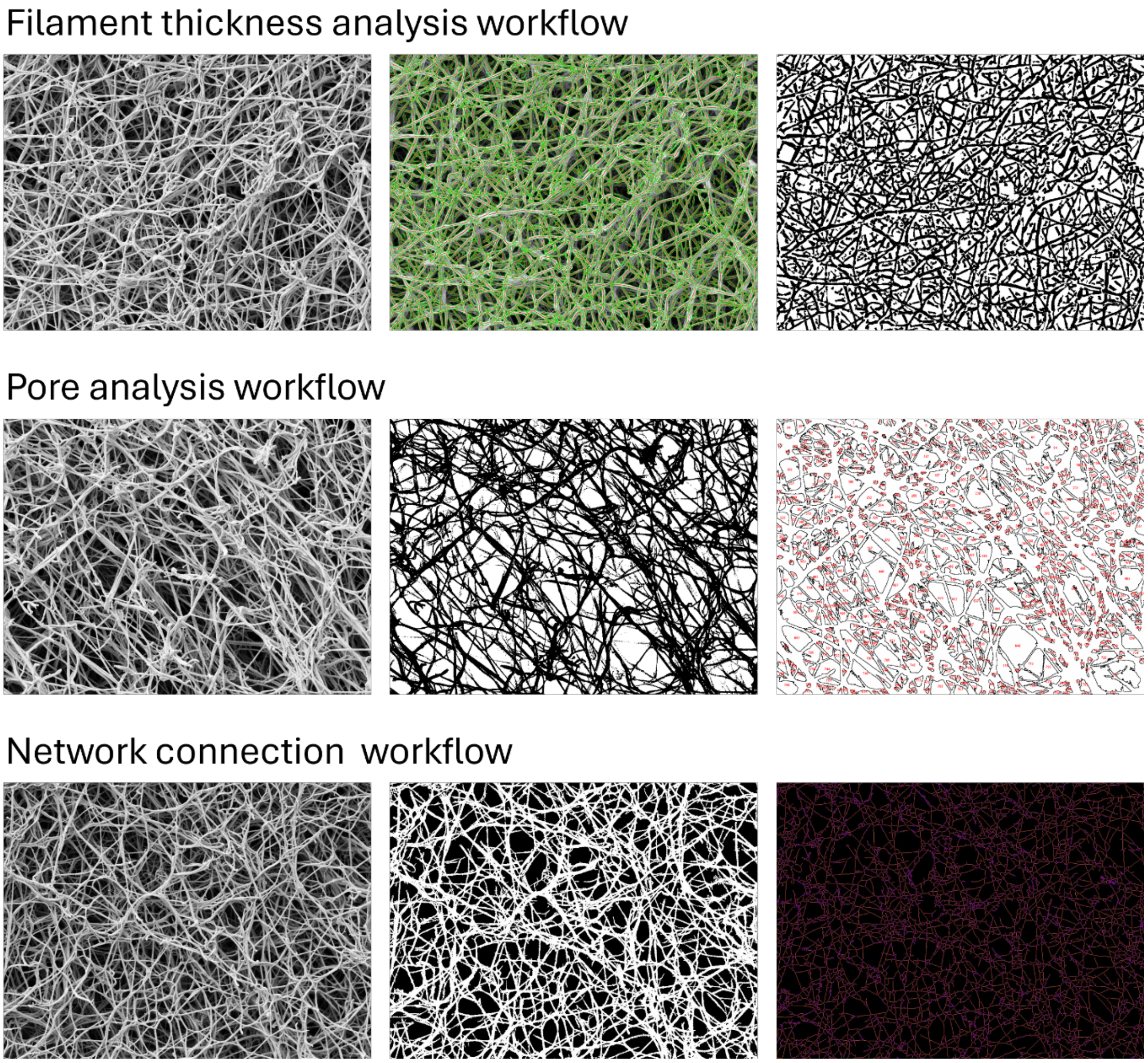

Figure S13: Representative images of SEM analysis workflows to generate information contained within Figure 3 and Supplementary Table 2. Top – Raw image (left; D5 gel from Figure 3) was contrast adjusted and used for ridge detection analysis tool (middle), with overlayed image of analysed fibres (right); Middle – Raw image (left; D5 gel repeat image; not shown in Figure 3) was threshold adjusted (middle) before quantification and sizing of pores through particle analysis tool (right); Bottom – Raw image (left; NC gel from Figure 3) was threshold adjusted and inverted (middle) before being skeletonized and assessed using the "Analyze Skeleton" tool (right).
